# Birth cohort-specific trends of sun-related behaviors among individuals from an international consortium of melanoma-prone families

**DOI:** 10.1186/s12889-021-10424-5

**Published:** 2021-04-23

**Authors:** John Charles A. Lacson, Shawn A. Zamani, Luis Alberto Ribeiro Froes, Nandita Mitra, Lu Qian, Scarlet H. Doyle, Esther Azizi, Claudia Balestrini, D. Timothy Bishop, William Bruno, Blanca Carlos-Ortega, Francisco Cuellar, Anne E. Cust, David E. Elder, Anne-Marie Gerdes, Paola Ghiorzo, Thais C. Grazziotin, Nelleke A. Gruis, Johan Hansson, Marko Hočevar, Veronica Höiom, Elizabeth A. Holland, Christian Ingvar, Gilles Landman, Alejandra Larre-Borges, Graham J. Mann, Montserrat Molgo, Luciana Facure Moredo, Håkan Olsson, Jacoba J. Out-Luiting, Barbara Perić, Dace Pjanova, Susana Puig, Julio Salas-Alanis, Helen Schmid, Karin A. W. Wadt, Julia A. Newton-Bishop, Peter A. Kanetsky, on behalf of the GenoMEL Study Group

**Affiliations:** 1 Department of Cancer Epidemiology, Division of Population Science, H. Lee Moffitt Cancer Center & Research Institute, 12902 Magnolia Dr., MRC 213, Tampa, FL 33612 USA; 2 Medical Research Council - Cancer Unit, Division of Clinical Medicine, University of Cambridge, Cambridge, Cambridgeshire UK; 3 Department of Pathology, School of Medicine, Universidade de São Paulo (USP), São Paulo, SP Brazil; 4 Department of Biostatistics, Epidemiology, and Informatics, University of Pennsylvania, Philadelphia, PA USA; 5 SWOG Statistics and Data Management Center, Public Health Sciences Division, Fred Hutchinson Cancer Research Center, Seattle, WA USA; 6 Department of Dermatology, Sheba Medical Center, Tel Hashomer, Israel; 7 Sackler Faculty of Medicine, Tel Aviv University, Tel Aviv, Israel; 8 Servicio de Dermatología, Hospital Dr. Sótero del Río, Santiago de Chile, Chile; 9 Institute of Medical Research at St James’s, University of Leeds, Leeds, UK; 10 Department of Internal Medicine and Medical Specialties, University of Genoa, Genoa, Italy; 11 Departamento de Ciencias Básicas, Escuela de Medicina Universidad de Monterrey, Monterrey, Mexico; 12 Melanoma Unit, Dermatology Department, Hospital Clinic Barcelona, Institut de Investigacions biomediques August Pi Sunyer (IDIBAPS), Universitat de Barcelona, Barcelona, Spain; 13 Consejo Nacional de Ciencia y Tecnología (CONACYT), Ciudad de México, Mexico; 14 Sydney School of Public Health, The University of Sydney, Sydney, New South Wales Australia; 15 Melanoma Institute Australia, The University of Sydney, Wollstonecraft, New South Wales Australia; 16 Department of Pathology and Laboratory Medicine, Hospital of the University of Pennsylvania, Philadelphia, PA USA; 17 Department of Clinical Genetics, University Hospital of Copenhagen, Copenhagen, Denmark; 18 Genetics of Rare Cancers, IRCCS Ospedale Policlinico San Martino, Genoa, Italy; 19 Pontifícia Universidade Católica do Rio Grande do Sul (PUCRS), Porto Alegre, RS Brazil; 20 Department of Dermatology, Leiden University Medical Centre, Leiden, The Netherlands; 21 Department of Oncology-Pathology, Karolinska Institutet, Stockholm, Sweden; 22 Institute of Oncology Ljubljana, Ljubljana, Slovenia; 23 Westmead Institute for Medical Research, The University of Sydney, Westmead, New South Wales Australia; 24 Departments of Clinical Sciences and Surgery, Lund University Hospital, Lund, Sweden; 25 Department of Pathology, Escola Paulista de Medicina, UNIFESP, São Paulo, Brazil; 26 Department of Pathology, AC Camargo Cancer Center, São Paulo, Brazil; 27 Unidad de Lesiones Pigmentadas, Cátedra de Dermatología, Hospital de Clínicas, Universidad de la República, Montevideo, Uruguay; 28 John Curtin School of Medical Research, Australian National University, Canberra, ACT Australia; 29 Pontificia Universidad Católica de Chile, Santiago de Chile, Chile; 30 Skin Cancer Department, AC Camargo Cancer Center, São Paulo, Brazil; 31 Latvian Biomedical Research and Study Centre, Riga, Latvia; 32 Centro de Investigacion Biomedica en Red de Enfermedades Raras (CIBERER), Instituto de Salud Carlos III, Barcelona, Spain; 33 Departament de Medicina, Universitat de Barcelona, Barcelona, Spain; 34 Dystrophic Epidermolysis Bullous Research Association Mexico, Monterrey, Mexico

**Keywords:** Trends, Sun-related behaviors, Sunscreen use, Sun exposure, Sunburn, Sunbed, Melanoma, High-risk families, Skin Cancer

## Abstract

**Background:**

Individuals from melanoma-prone families have similar or reduced sun-protective behaviors compared to the general population. Studies on trends in sun-related behaviors have been temporally and geographically limited.

**Methods:**

Individuals from an international consortium of melanoma-prone families (GenoMEL) were retrospectively asked about sunscreen use, sun exposure (time spent outside), sunburns, and sunbed use at several timepoints over their lifetime. Generalized linear mixed models were used to examine the association between these outcomes and birth cohort defined by decade spans, after adjusting for covariates.

**Results:**

A total of 2407 participants from 547 families across 17 centers were analyzed. Sunscreen use increased across subsequent birth cohorts, and although the likelihood of sunburns increased until the 1950s birth cohort, it decreased thereafter. Average sun exposure did not change across the birth cohorts, and the likelihood of sunbed use increased in more recent birth cohorts. We generally did not find any differences in sun-related behavior when comparing melanoma cases to non-cases. Melanoma cases had increased sunscreen use, decreased sun exposure, and decreased odds of sunburn and sunbed use after melanoma diagnosis compared to before diagnosis.

**Conclusions:**

Although sunscreen use has increased and the likelihood of sunburns has decreased in more recent birth cohorts, individuals in melanoma-prone families have not reduced their overall sun exposure and had an increased likelihood of sunbed use in more recent birth cohorts. These observations demonstrate partial improvements in melanoma prevention and suggest that additional intervention strategies may be needed to achieve optimal sun-protective behavior in melanoma-prone families.

**Supplementary Information:**

The online version contains supplementary material available at 10.1186/s12889-021-10424-5.

## Background

The incidence of cutaneous malignant melanoma has been increasing over the past 50 years in populations that are predominantly fair-skinned [1]. Although rates have stabilized recently in several parts of the world including Australasia and North America, they continue to rise in most European and South American countries [1–3]. Exposure to ultraviolet radiation, especially intermittent sun exposure resulting in sunburn, is the main environmental factor associated with increased melanoma risk [4].

In the general population, sun-protective behavior has improved, evidenced by increased sunscreen use and increased sun protection factor (SPF) [5–8]. However, among individuals with a family history of melanoma, who have double to quadruple the risk of developing melanoma compared to the general population [9], sun-seeking and tanning behaviors are still prevalent [10]. In addition, sun-protective behaviors are suboptimal among children and adolescents who have a family history of melanoma [10–13] and among adolescents in the general population [14, 15]. These deficits are notable because ultraviolet radiation exposure during childhood and adolescence is a strong determinant of melanoma risk [16, 17].

Previous studies on sun-protective behaviors in the general population were limited to a specific geographic region, examined trends only within a single or a few decades, or did not examine behaviors throughout the lifetime of participants [5, 6, 14, 18–25]. Studies of sun-related behaviors among individuals with a family history of melanoma mainly focused on recent exposure and rarely describe trends over time [10, 12, 26–28].

We report results from a large international study of melanoma-prone families, in which participants were queried about sun-related behaviors at several points over their lifetime, including childhood and adolescence. We characterize trends in sun-related behavior over most of the twentieth century by birth cohort and compare between melanoma cases and non-cases. Among cases, we also compare these behaviors before and after melanoma diagnosis.

## Methods

### Study population

The Melanoma Genetics Consortium, GenoMEL, recruited individuals from melanoma-prone families at 27 centers across the globe. Our study sample included participants from 17 GenoMEL centers in Europe, North and South America, Australia, and the Middle East. Melanoma-prone families were defined by the presence of three or more melanoma cases among blood relatives, or two or more cases in first-degree relatives. Personal history of melanoma was self-reported or verified from pathology report, cancer registry, clinical notation, or death certificate. Participants were mailed a questionnaire that solicited information on demographics, personal history of melanoma, phenotypic variables, and a personal residence calendar that captured yearly information on location of residence, school and/or work, and the average number of days per week spent at school and/or work (Additional file 1). A subsequent questionnaire administered by GenoMEL research staff recorded details on personal sun exposure and sun-protective behaviors at multiple time points across the life course, using the residence and school/work calendar as memory prompts (Additional file 2). This instrument was adapted from one used in several epidemiological studies of melanoma and has been previously validated [29, 30].

### Demographics and phenotypic covariates

Information on sex and date of birth were obtained. Individuals were grouped into decade birth cohorts starting with the 1910s and ending with the 1980s. We combined the participants born in 1910s (*n* = 15) with those born in 1920s (*n* = 136). Participants born in the 1990s (*n* = 29) or 2000s (*n* = 1) were excluded due to limited lifecourse information. Phenotypic information was collected on hair color (red, blonde, light brown, dark brown, black), eye color (blue, brown/black, other), and skin complexion (very fair, fair, olive, brown, black, other). Participants reported burn severity after an hour of mid-day summer sun exposure without protection (severe sunburn with skin blistering, painful sunburn with skin peeling, mildly sunburned with some tanning, or no sunburn with brown tan), and tanning after repeated sun exposure without protection (deep brown tan, moderate tan, mild tan, or no tan/only freckling). Additionally, participants reported face freckling in childhood and as an adult (none, very few, few, some, many, very many), and the number of moles on the body (none, few, many, very many).

### Sunscreen use, sun exposure, sunburn, and sunbed use

Information on sunscreen use, sun exposure, and sunburns was obtained at anchor years of 10, 15, 20, 30, 40 years old, and the year before the completion date of the survey questionnaire. For each anchor year, participants were asked about the frequency of sunscreen use while exposed to sunlight on a five-point scale (1 - never or hardly ever, 2 - less than half the time, 3 - about half the time, 4 - more than half the time, 5 - always or almost always). For participants who reported sunscreen use, use of sunscreen with a SPF of 8 or higher (yes, no) was assessed. A SPF-weighted sunscreen variable was formulated by assigning individuals who reported using SPF less than 8 to having never used sunscreen during that timepoint.

At each anchor year, participants were asked to report personal outdoor sun exposure and the number of painful sunburns that lasted for two or more days. We obtained detailed information on number of hours spent outside between 9 AM and 5 PM during weekdays, weekends, and holidays, separately for warmer and colder months. Holidays were defined as days spent away from school or work.

Daily average sun exposure (time spent outside in hours) was calculated separately for weekdays, weekends, and holidays, combining responses from warmer and colder months within each anchor year. To calculate overall sun exposure, a time-weighted integration of hours spent outdoors was calculated based on 5 days for weekdays and 2 days for weekend days during work and school weeks, and 7 days for holiday weeks over the 26 weeks for warmer and colder months, respectively. Hours of warmer and colder months were then averaged to generate outdoor hours for the entire year. To calculate lifetime average sun exposure, data from each anchor year were assigned to a specific period of life: anchor year 10 was assigned to ages 5 to 12, anchor year 15 to ages 13 to 17, anchor year 20 to ages 18 to 24, anchor year 30 to ages 25 to 34, anchor year 40 to ages 35 to 44, and the last anchor year to ages 45 and above. Sun exposure measurements were then averaged over the number of years recorded for each individual. The following imputation scheme was used for missing values: if either the first or last anchor year was missing, the closest non-missing value was carried backward or forward. If a missing anchor year was between two non-missing values, the average of the two adjacent non-missing values was used. Imputation was done separately for weekdays, weekends, and holidays, within warmer and colder months.

Participants reported sunlamp or sunbed use on more than one occasion (ever, never). This question was asked only once covering the entire life course and not at each anchor year. Participants who responded an ever-use of sunlamps or sunbeds were asked about their age at first- and last-use.

### Statistical analyses

#### Factor analysis of phenotypic variables

Due to the high covariance of phenotypic variables, factor analysis was performed. Following components extraction using principal axis method with Varimax (orthogonal) rotation, three meaningful factors emerged and were retained for rotation. Each of these components had an eigenvalue > 1.00 and have a cumulative percent variance > 70%. Each variable was loaded on a component if the rotated factor pattern loading was greater than 0.40 for that factor and less than 0.40 for the other two factors. Using this scheme, burnability, tanability, skin complexion loaded on the first factor; hair color, eye color, extent of body moles loaded on the second; and childhood and adulthood face freckles loaded on the third. Factor-based scores were calculated per participant. Factors were modeled in a confirmatory factor analysis and robust maximum likelihood estimation yielded a good fit with the model (RMSEA = 0.05; CFI = 0.97; SRMR = 0.03). We tested for heterogeneity of trend by pre- vs. post-diagnosis by introducing an interaction term between diagnostic period and birth cohort. All statistical analyses were conducted using SAS v. 9.4 (Statistical Analysis System, RRID:SCR_008567).

#### Life course and age group analyses

All statistical analyses were conducted using SAS v. 9.4 (Statistical Analysis System, RRID:SCR_008567). Predicted population marginal means were estimated using SAS GLIMMIX procedure; separate models were used for sunscreen use (including SPF-weighted use), hours of sun exposure, ever having sunburns, or ever use of sunbeds as the outcome and birth cohort as the predictor (categorical) variable. We conducted trend analyses by coding birth cohort as an ordinal variable in the model. All models included random effects to account for clustering within family, and we used empirical (sandwich) variance estimators. In the models, sunscreen data were assumed to follow a gamma distribution, and sun exposure data were assumed to follow a normal distribution. Due to sparse data, we modeled sunburn data as a binomial outcome. Analyses of sunscreen use commenced with the 1950 birth cohort, which corresponds to the decade sunscreen became commercially available [31].

All models were adjusted for sex, center (grouped by latitude: high northern, mid-high northern, mid northern, southern), and the three factor variables for phenotype. We conducted analyses for the entire life course and within three life periods: 10 years old or younger, 11 to 20 years, and after 20 years old. Life course models and models for after 20 years old also included a covariate capturing number of years unaffected with melanoma, which was equal to the age at diagnosis for cases, or to the age at interview for non-cases.

#### Analyses stratified by melanoma status, and before and after diagnosis

We used the same models as above to obtain predicted population marginal means and perform tests of trends after stratifying by melanoma status. We tested for the difference of each outcome variable by melanoma status across the birth cohorts by adding melanoma status as a covariate to the full model. Tests of heterogeneity of trend were conducted by adding an interaction term between birth cohort and melanoma status.

For melanoma cases, all outcome variables were further defined by whether they occurred before or after diagnosis and were compared using the SAS GLIMMIX procedure. Random effects were included in the model to account for clustering of individuals within families. Because of implicit individual-matching across the pre- and post-diagnostic periods, other covariates were not included in these models. We tested for heterogeneity of trend by pre- vs. post-diagnosis by introducing an interaction term between diagnostic period and birth cohort.

## Results

A total of 2407 participants from 547 families across 17 centers were recruited (Table 1). The mean age of participants was 52.9 years old (SD = 16), and 59% were female (Table 2). The majority (60%) of participants were born in the 1940s, 1950s, or 1960s, with approximately 20% in each decade. Almost half (47.8%) of participants reported at least one primary cutaneous melanoma.

**Table 1:** Number of families, individual, and melanoma affected or unaffected individuals per GenoMEL center

Latitude	Center	Families	Individuals	Unaffected	Affected
High northern	Copenhagen, DK	26	52	0	52
	Lund, SE	6	41	25	16
	Stockholm, SE	22	125	89	36
Mid-high northern	Leeds, UK	93	303	139	164
	Leiden, NL	47	431	309	122
	Ljubljana, SI	6	16	2	14
	Riga, LV	3	8	5	3
Mid northern	Barcelona, ES	35	147	91	56
	Genoa, IT	25	79	25	54
	Philadelphia, US	31	95	31	64
	Tel Aviv, IL	31	127	66	61
Southern	Mexico City, MX	2	9	5	4
	Montevideo, UY	9	55	42	13
	Porto Alegre, BR	17	40	16	24
	Santiago, CL	4	10	2	8
	São Paulo, BR	15	65	36	29
	Sydney, AU	175	804	374	430
**Total**	**17**	**547**	**2407**	**1257**	**1150**

**Table 2:** Characteristics of GenoMEL participants (*N* = 2407)

Variable	Individuals, ***n*** (%)	
***Age at Interview***		
Mean (SD)	52.9	(16)
< 20	16	(0.7)
20–30	182	(7.6)
30–40	340	(14.1)
40–50	478	(19.9)
50–60	514	(21.4)
60–70	459	(19.1)
70–80	319	(13.3)
80+	99	(4.2)
***Birth decade***		
1910–1929	147	(6.1)
1930–1939	351	(14.6)
1940–1949	493	(20.5)
1950–1959	501	(20.8)
1960–1969	482	(20.0)
1970–1979	292	(12.1)
1980–1989	141	(5.9)
***Sex***		
Female	1421	(59.0)
Male	986	(41.0)
***Hair color***		
Red	279	(11.6)
Blonde or fair	848	(35.2)
Brown	1164	(48.4)
Black	104	(4.3)
Missing	12	(0.5)
***Eye color***		
Blue	874	(36.3)
Gray, hazel or green	1062	(44.1)
Brown or black	464	(19.3)
Missing	7	(0.3)
***Skin complexion***		
Very fair	490	(20.4)
Fair	1699	(70.6)
Darker^a^	205	(8.5)
Missing	13	(0.5)
***Burnability***		
Severe burn, blister	194	(8.1)
Painful burn, peel	1032	(42.9)
Mild burn, some tan	959	(39.8)
No burn, brown tan	165	(6.9)
Missing	57	(2.4)
***Tanability***		
No tan or get freckled	266	(11.1)
Moderate tan	1045	(43.4)
Mild or occasional tan	761	(31.6)
Deep tan	272	(11.3)
Missing	63	(2.6)
***Childhood face freckles***		
Very many	40	(1.7)
Many	176	(7.3)
Some	304	(12.6)
Few	464	(19.3)
Very few	665	(27.6)
None	723	(30.0)
Missing	35	(1.5)
***Adulthood face freckles***		
Very many	23	(1.0)
Many	77	(3.2)
Some	211	(8.8)
Few	343	(14.3)
Very few	798	(33.2)
None	916	(38.1)
Missing	39	(1.6)
***Extent of body moles***		
Many	530	(22.0)
Some	916	(38.1)
Few	760	(31.6)
None	167	(6.9)
Missing	34	(1.4)
***Melanoma history***		
Affected	1150	(47.8)
Unaffected	1257	(52.2)
***Center latitude***		
High northern	218	(9.1)
Mid-high northern	758	(31.5)
Mid northern	448	(18.6)
Southern	983	(40.8)

^a^ Darker skin includes olive, brown, black and other

### Sunscreen use

**Fig. 1:**
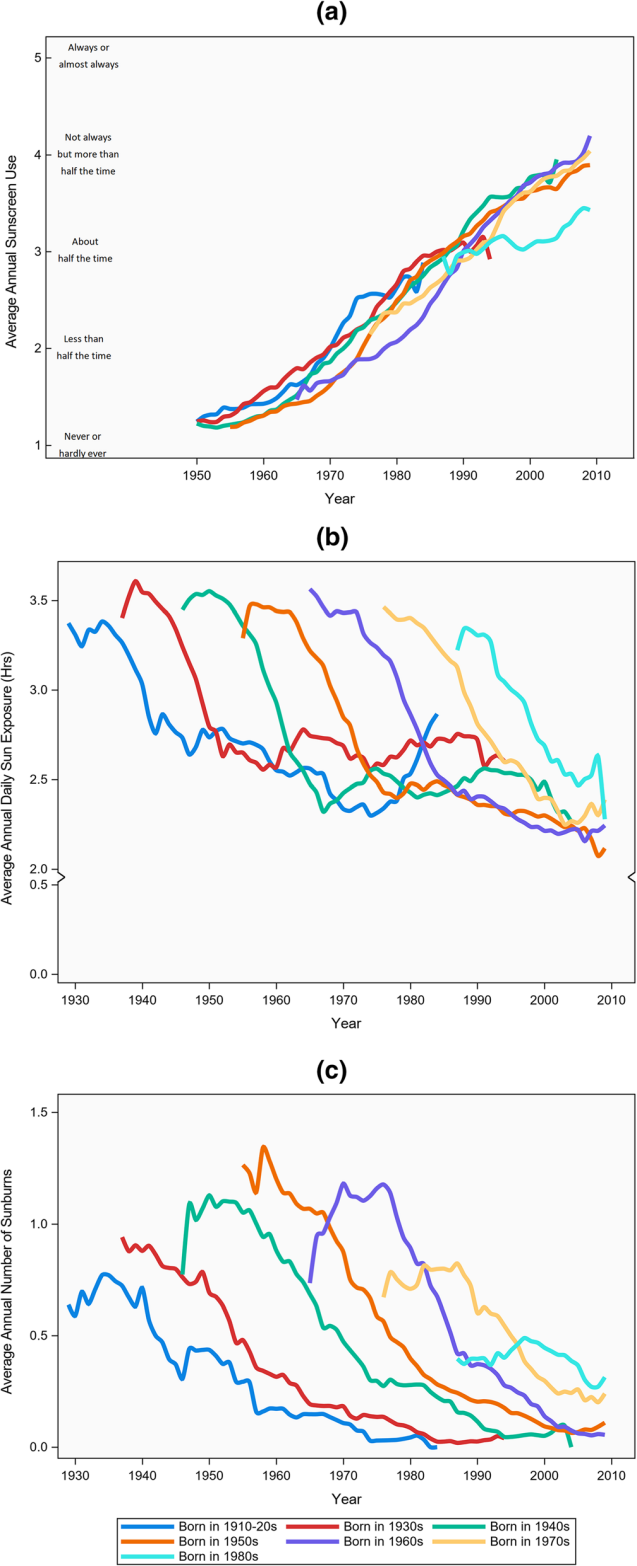
Trends of average annual sunscreen use **a**, average annual daily sun exposure (in hours) **b**, and average annual frequency of sunburns **c** over time and by birth cohort among GenoMEL participants

We noted a clear secular trend in average reported frequency of sunscreen use with nearly identical increases over time across all birth cohorts (Fig. 1a). Sunscreen use increased as each birth cohort aged, and more recent cohorts had increased sunscreen use at younger ages compared to earlier birth cohorts. Results were similar after adjusting for SPF (data not shown).

Adjusted average lifetime sunscreen use increased by birth cohort (per birth cohort β = 0.08, *P* = 0.004), and this trend strengthened after weighting by SPF (β = 0.11, *P* = 0.0003, Table 3). This trend of increasing sunscreen with subsequent birth cohort was evident within each life period, within strata defined by melanoma status, and before and after melanoma diagnosis. We found no difference or heterogeneity in trends when comparing cases and non-cases. Cases had higher average sunscreen use (adjusted mean difference (AMD) = 1.36, *P* < 0.0001) after diagnosis than before diagnosis. All results were similar after weighting by SPF.

**Table 3:** Trends in sunscreen use and sun exposure among GenoMEL participants

Birth decade	***n***	(%)	Sunscreen use		Daily sun exposure (hours)			
			Overall	SPF-Weighted	Overall	During weekdays	During weekends	During holidays
***Overall***^***a***^								
1910–1929	147	(6.4)	2.40	2.29	2.22	2.04	2.67	1.43
1930–1939	351	(15.2)	2.59	2.47	2.59	2.35	3.17	2.89
1940–1949	493	(21.4)	2.57	2.39	2.52	2.19	3.32	2.73
1950–1959	501	(21.7)	2.59	2.37	2.36	1.98	3.27	2.79
1960–1969	482	(20.9)	2.69	2.47	2.35	1.97	3.25	2.89
1970–1979	292	(12.6)	2.87	2.55	2.45	2.13	3.19	2.82
1980–1989	141	(6.1)	2.92	2.76	2.44	2.13	3.17	2.99
Total	2407							
*Beta*^*b*^			0.08	0.11	−0.01	−0.04	0.04	0.05
*P*_*trend*_^a^			*0.004*	*0.0003*	*0.64*	*0.26*	*0.26*	*0.38*
***10 years old or younger***^***c***^								
1910–1929	147	(6.4)	*	*	3.40	2.38	4.30	4.80
1930–1939	351	(15.2)	*	*	3.48	2.42	4.61	4.83
1940–1949	493	(21.4)	1.19	1.13	3.56	2.47	4.64	4.97
1950–1959	501	(21.7)	1.38	1.23	3.44	2.34	4.54	4.79
1960–1969	482	(20.9)	1.89	1.61	3.42	2.39	4.46	4.66
1970–1979	292	(12.6)	2.47	2.18	3.43	2.41	4.36	4.64
1980–1989	141	(6.1)	3.18	2.99	3.19	2.37	3.95	4.24
Total	2407							
*Beta*^*b*^			0.41	0.34	−0.03	−0.01	−0.07	−0.08
*P*_*trend*_^*c*^			*< 0.0001*	*< 0.0001*	*0.04*	*0.65*	*0.003*	*0.0002*
***11 to 20 years old***^***c***^								
1910–1929	147	(6.4)	*	*	2.79	2.22	3.68	3.29
1930–1939	351	(15.2)	1.32	1.21	2.86	2.20	3.93	3.48
1940–1949	493	(21.4)	1.37	1.23	2.88	2.14	3.92	3.83
1950–1959	501	(21.7)	1.64	1.40	2.85	2.06	3.83	3.94
1960–1969	482	(20.9)	2.10	1.80	2.93	2.13	3.87	4.07
1970–1979	292	(12.6)	2.83	2.42	3.01	2.26	3.79	4.05
1980–1989	141	(6.1)	3.16	2.99	2.73	2.09	3.41	3.53
Total	2407							
*Beta*^*b*^			0.33	0.27	0.01	−0.01	−0.04	0.09
*P*_*trend*_^*c*^			*< 0.0001*	*< 0.0001*	*0.37*	*0.76*	*0.04*	*0.0001*
***After 20 years old***^***a***^								
1910–1929	147	(6.4)	2.53	2.43	2.22	2.05	2.65	1.41
1930–1939	351	(15.2)	2.79	2.66	2.62	2.38	3.17	2.94
1940–1949	493	(21.4)	2.98	2.78	2.53	2.21	3.30	2.78
1950–1959	501	(21.7)	3.10	2.87	2.37	1.98	3.28	2.83
1960–1969	482	(20.9)	3.20	3.02	2.35	1.97	3.23	2.87
1970–1979	292	(12.6)	3.30	3.05	2.49	2.17	3.17	2.74
1980–1989	141	(6.1)	2.93	2.76	2.51	2.15	3.18	2.97
Total	2407							
*Beta*^*b*^			0.13	0.10	−0.01	−0.03	0.03	0.03
*P*_*trend*_^*a*^			*0.0002*	*0.002*	*0.83*	*0.31*	*0.33*	*0.64*
***Non-cases***^***c***^								
1910–1929	77	(6.1)	1.71	1.60	2.59	2.50	2.84	1.39
1930–1939	164	(13.0)	2.15	2.03	2.68	2.44	3.25	2.90
1940–1949	207	(16.5)	2.34	2.14	2.71	2.40	3.50	2.98
1950–1959	268	(21.3)	2.38	2.13	2.50	2.14	3.36	2.98
1960–1969	242	(19.3)	2.76	2.47	2.49	2.11	3.38	3.08
1970–1979	189	(15.0)	2.97	2.68	2.34	1.98	3.17	3.03
1980–1989	110	(8.8)	3.26	3.11	2.48	2.15	3.24	3.21
Total	1257							
*Beta*^*b*^			0.23	0.24	−0.06	− 0.09	0.01	0.08
*P*_*trend*_^c^			*< 0.0001*	*< 0.0001*	*0.02*	*0.001*	*0.73*	*0.10*
***Melanoma Cases***^***a***^								
1910–1929	70	(6.1)	2.70	2.57	2.04	1.81	2.59	1.82
1930–1939	187	(16.3)	2.69	2.55	2.63	2.41	3.15	3.10
1940–1949	286	(24.9)	2.64	2.46	2.39	2.08	3.18	2.63
1950–1959	233	(20.3)	2.83	2.65	2.20	1.80	3.16	2.57
1960–1969	240	(20.9)	2.75	2.59	2.13	1.71	3.09	2.59
1970–1979	103	(9.0)	3.13	2.74	2.52	2.23	3.22	2.44
1980–1989	31	(2.7)	2.92	2.71	1.91	1.47	2.94	2.45
Total	1150							
*Beta*^*b*^			0.07	0.10	−0.04	−0.07	0.04	−0.07
*P*_*trend*_^a^			*0.08*	*0.02*	*0.33*	*0.11*	*0.25*	*0.43*
*Adjusted Mean Difference*^*a*^			0.008	0.06	−0.11	−0.12	− 0.07	−0.46
*P*_*AMD*_^*a*^			0.91	0.36	0.11	0.15	0.39	0.002
*P*_*heterogeneity of trend*_^*a*^			0.69	0.69	0.59	0.85	0.31	0.51
***Pre-Diagnosis***^***c***^								
1910–1929	70	(6.1)	2.10	1.94	2.31	1.84	3.07	3.58
1930–1939	187	(16.3)	2.10	1.92	2.83	2.42	3.52	3.78
1940–1949	285	(24.9)	2.07	1.85	2.70	2.12	3.63	3.98
1950–1959	232	(20.3)	2.35	2.14	2.64	1.98	3.58	3.88
1960–1969	239	(20.9)	2.50	2.33	2.72	1.98	3.65	3.96
1970–1979	101	(8.8)	3.15	2.71	3.02	2.26	3.73	4.13
1980–1989	31	(2.7)	3.11	2.86	2.78	2.05	3.57	3.75
Total	1145							
*Beta*^*b*^			0.18	0.16	0.05	−0.03	0.06	0.05
*P*_*trend*_^*c*^			*< 0.0001*	*< 0.0001*	*0.02*	*0.23*	*0.01*	*0.07*
***Post-Diagnosis***^***c***^								
1910–1929	70	(6.1)	2.79	2.73	1.92	1.86	2.11	2.48
1930–1939	186	(16.4)	3.16	3.07	2.62	2.53	2.86	3.43
1940–1949	283	(25.0)	3.71	3.64	2.42	2.20	2.93	2.74
1950–1959	231	(20.4)	4.08	4.02	2.10	1.68	3.05	3.08
1960–1969	234	(20.6)	3.91	3.87	2.08	1.70	2.98	2.78
1970–1979	101	(8.9)	4.04	3.91	2.63	2.31	3.33	3.54
1980–1989	29	(2.6)	3.47	3.40	2.28	1.77	3.23	3.09
Total	1134							
*Beta*^*b*^			0.24	0.24	−0.02	−0.10	0.13	0.04
*P*_*trend*_^*c*^			*< 0.0001*	*< 0.0001*	*0.43*	*0.003*	*< 0.0001*	*0.62*
*Adjusted Mean Difference*^*d*^			1.36	1.5	−0.41	− 0.08	− 0.63	−0.88
*P*_*AMD*_^*d*^			< 0.0001	< 0.0001	< 0.0001	0.04	< 0.0001	< 0.0001
*P*_*heterogeneity of trend*_^*d*^			0.04	0.001	0.10	< 0.0001	0.0004	0.94

* Sunscreen was not widely available until the 1950s; analyses were limited to 1950s and afterwards

^a^ Model adjusted for sex, phenotype factor variables, center geography, years unaffected, and clustered by family

^b^ Betas represent the average change per unit increase in birth decade, after adjusting for covariates

^c^ Model adjusted for sex, phenotype factor variables, center geography, and clustered by family

^d^ Model adjusted for repeated individual data clustered by family

### Sun exposure

There was no apparent difference in unadjusted average sun exposure across birth cohorts or over time period, but sun exposure decreased as each cohort aged (Fig. 1b). We did not observe a trend in the adjusted lifetime average daily sun exposure with increasing birth cohort overall, or during weekdays, weekends, or holidays (Table 3). At 10 years of age or younger, average daily sun exposure during weekends (β = − 0.07, *P*_*trend*_ = 0.003), holidays (β = − 0.08, *P*_*trend*_ = 0.0002), and overall (β = − 0.03, *P*_*trend*_ = 0.04) decreased in more recent birth cohorts. This trend remained for weekends (β = − 0.07, *P*_*trend*_ = 0.04), although sun exposure increased during holidays (β = 0.09, *P*_*trend*_ = 0.0001) when participants were between 11 to 20 years old. We did not observe a trend in average sun exposure by birth cohort when participants were older than 20 years. Cases had less sun exposure during holidays (AMD = − 0.46 h, *P* = 0.002) compared to non-cases, otherwise there were no differences or heterogeneity in trend for sun exposure between cases and non-cases.

After diagnosis, cases had lower average daily sun exposure overall (AMD = − 0.41 h, *P* < 0.0001), which was primarily driven by differences during weekends (AMD = − 0.63 h, *P* < 0.0001), holidays (AMD = − 0.88 h, *P* < 0.0001), and to a lesser extent, weekdays (AMD = − 0.08 h, *P* = 0.04), compared to before diagnosis. Although there was no heterogeneity of trend overall and during holidays, there was an accelerated decrease across the birth cohorts in sun exposure during weekdays (*P* < 0.0001) and weekends (*P* = 0.0004) after diagnosis compared to before diagnosis.

### Sunburns

Within birth cohorts, average number of sunburns occurred more frequently at younger ages and decreased with age (Fig. 1c). The frequency of early life sunburns increased until the 1950s birth cohort and then decreased, with the lowest frequency of sunburns at younger ages observed in the most recent (1980s) cohort. Similarly, frequency of sunburns increased and peaked in 1960 and decreased thereafter.

A total of 1598 (66.4%) respondents reported ever being sunburned (Table 4). We did not observe a trend of odds of ever being sunburned with increasing birth cohort or within the three life periods after adjustment. Cohort-specific odds ratios tended to increase until the 1950s cohort and decrease thereafter. This inverted U-shape trend was verified in models containing a quadratic birth cohort term (data not shown). There was no difference in odds of ever being sunburned between melanoma cases and non-cases (OR = 1.12, 95%CI: 0.88 to 1.42), nor was there heterogeneity of trend. Cases were less likely to have sunburns after diagnosis with melanoma than before (OR = 0.04, 95%CI: 0.04 to 0.05). The trend in odds of ever being sunburned across subsequent birth cohorts was (inverted) U-shaped before diagnosis, in contrast to an increasing linear trend seen after diagnosis.

**Table 4:** Adjusted odds ratios and 95% confidence intervals for the association between birth decade and history of sunburns and sunbed use among GenoMEL participants

Birth decade	Sunburn					Sunbed use				
	***n***	Never ***n*** (%)	Ever ***n*** (%)	OR	95% CI	***n***	Never ***n*** (%)	Ever ***n*** (%)	OR	95% CI
***Overall***^***a***^										
1910–1929	147	63 (42.9)	84 (57.1)	1.00	REF	144	130 (90.3)	14 (9.7)	1.00	REF
1930–1939	351	147 (41.9)	204 (58.1)	1.07	(0.70 1.63)	349	292 (83.7)	57 (16.3)		
1940–1949	493	142 (28.8)	351 (71.2)	2.03	(1.28 3.22)	492	366 (74.4)	126 (25.6)	2.29	(1.48 3.53)
1950–1959	501	132 (26.4)	369 (73.7)	2.20	(1.39 3.48)	497	353 (71.0)	144 (29.0)	3.05	(1.94 4.79)
1960–1969	482	137 (28.4)	345 (71.6)	1.92	(1.10 3.35)	476	294 (61.8)	182 (38.2)	6.12	(3.78 9.89)
1970–1979	292	113 (38.7)	179 (61.3)	1.12	(0.60 2.11)	289	173 (59.9)	116 (40.1)	6.36	(3.53 11.44)
1980–1989	141	75 (53.2)	66 (46.8)	0.69	(0.32 1.46)	138	94 (68.1)	44 (31.9)	4.38	(2.12 9.07)
Total	2407	809 (33.6)	1598 (66.4)			2385	1702 (71.4)	683 (28.6)		
*Per Cohort OR*				0.96	(0.86 1.08)				1.44	(1.28 1.62)
*P*_*trend*_				0.52					< 0.0001	
***10 years old or younger***^***b***^										
1910–1929	140	93 (66.4)	47 (33.6)	1.00	REF	*				
1930–1939	331	212 (64.0)	119 (36.0)	1.24	(0.78 1.99)	*				
1940–1949	467	270 (57.8)	197 (42.2)	1.68	(1.07 2.62)	*				
1950–1959	481	253 (52.6)	228 (47.4)	2.05	(1.32 3.17)	*				
1960–1969	464	276 (59.5)	188 (40.5)	1.57	(0.97 2.54)	*				
1970–1979	283	191 (67.5)	92 (32.5)	1.04	(0.62 1.73)	*				
1980–1989	139	108 (77.7)	31 (22.3)	0.71	(0.39 1.29)	*				
Total	2305	1403 (58.3)	902 (41.7)							
*Per Cohort OR*				0.96	(0.90 1.02)					
*P*_*trend*_				0.21						
***11 to 20 years old***^***b***^										
1910–1929	147	68 (46.3)	79 (53.7)	1.00	REF	*				
1930–1939	349	160 (45.9)	189 (54.1)	1.07	(0.71 1.61)	*				
1940–1949	493	165 (33.5)	328 (66.5)	1.88	(1.24 2.85)	*				
1950–1959	501	155 (30.9)	346 (69.1)	2.10	(1.42 3.12)	*				
1960–1969	479	146 (30.5)	333 (69.5)	2.27	(1.47 3.51)	*				
1970–1979	292	119 (40.8)	173 (59.2)	1.36	(0.87 2.13)	*				
1980–1989	141	76 (53.9)	65 (46.1)	0.94	(0.56 1.57)	*				
Total	2402	889 (36.9)	1513 (63.1)							
*Per Cohort OR*				1.04	(0.97 1.11)					
*P*_*trend*_				0.30						
***After 20 years old***^***a***^										
1910–1929	147	114 (77.6)	33 (22.4)	1.00	REF					
1930–1939	351	242 (69.0)	109 (31.0)	1.71	(1.04 2.83)	*				
1940–1949	491	278 (56.6)	213 (43.4)	3.13	(1.89 5.20)	*				
1950–1959	501	283 (56.5)	218 (43.5)	3.29	(1.97 5.49)	*				
1960–1969	482	297 (61.6)	185 (38.4)	2.82	(1.60 4.96)	*				
1970–1979	291	197 (67.7)	94 (32.3)	2.31	(1.24 4.29)	*				
1980–1989	106	82 (77.4)	24 (22.6)	1.47	(0.67 3.22)	*				
Total	2369	1493 (63.0)	876 (37.0)			*				
*Per Cohort OR*				1.05	(0.96 1.14)					
*P*_*trend*_				0.33						
***Non-cases***^***b***^										
1910–1929	77	36 (46.8)	41 (53.3)	1.00	REF	74	67 (90.5)	7 (9.5)	1.00	REF
1930–1939	164	71 (43.3)	93 (56.7)	1.08	(0.60 1.95)	163	199 (84.0)	39 (16.0)		
1940–1949	207	78 (37.7)	129 (62.3)	1.64	(0.94 2.86)	207	141 (68.1)	66 (31.9)	2.06	(1.19 3.55)
1950–1959	268	74 (27.6)	194 (72.4)	2.60	(1.47 4.60)	266	178 (66.9)	88 (33.1)	2.11	(1.20 3.55)
1960–1969	242	80 (33.1)	162 (66.9)	2.06	(1.17 3.65)	240	145 (60.4)	95 (39.6)	3.20	(1.89 5.41)
1970–1979	189	81 (42.9)	108 (57.1)	1.19	(0.65 2.15)	189	110 (58.2)	79 (41.8)	3.20	(1.81 5.63)
1980–1989	110	60 (54.6)	50 (45.4)	0.86	(0.44 1.68)	108	72 (66.7)	36 (33.3)	2.03	(1.09 3.77)
Total	1257	480 (38.2)	777 (61.8)			1247	845 (67.8)	402 (32.2)		
*Per Cohort OR*				0.99	(0.91 1.08)				1.19	(1.09 1.30)
*P*_*trend*_				0.78					0.0002	
***Melanoma cases***^***a***^										
1910–1929	70	27 (38.6)	43 (61.4)	1.00	REF	70	63 (90.0)	7 (10.0))	1.00	REF
1930–1939	187	76 (40.6)	111 (59.4)	1.10	(0.58 2.11)	186	160 (86.0)	26 (14.0))		
1940–1949	286	64 (22.4)	222 (77.6)	2.90	(1.48 5.66)	285	225 (79.0)	60 (21.0)	2.06	(1.17 3.64)
1950–1959	233	58 (24.9)	175 (75.1)	2.44	(1.27 4.70)	231	175 (75.8)	56 (24.2)	2.73	(1.47 5.04)
1960–1969	240	57 (23.8)	183 (76.2)	2.68	(1.21 5.93)	236	149 (63.1)	87 (36.9)	6.46	(3.47 12.05)
1970–1979	103	32 (31.1)	71 (68.9)	1.89	(0.79 4.50)	100	63 (63.0)	37 (37.0)	5.80	(2.60 12.93)
1980–1989	31	15 (48.4)	16 (51.6)	0.92	(0.29 2.92)	30	22 (73.3)	8 (26.7)	2.83	(0.81 9.94)
Total	1150	329 (28.6)	821 (71.4)			1138	857 (75.3)	281 (24.7)		
*Per Cohort OR*				1.00	(0.99 1.02)				1.46	(1.24 1.71)
*P*_*trend*_				0.29					< 0.0001	
*OR*_*cases* vs *non-cases*_				1.12	(0.88 1.42)				0.89	(0.67 1.16)
*P*_*heterogeneity of trend*_				0.21					0.21	
***Pre-Diagnosis***^***b***^										
1910–1929	70	27 (38.6)	43 (61.4)	1.00	REF	70	65 (92.9)	5 (7.1)	1.00	REF
1930–1939	187	77 (41.2)	110 (58.8)			183	163 (89.1)	20 (10.9)		
1940–1949	285	63 (22.1)	222 (77.9)	2.62	(1.69 4.05)	285	237 (83.2)	48 (16.8)	1.86	(1.05 3.29)
1950–1959	232	57 (24.6)	175 (75.4)	2.16	(1.44 3.25)	226	182 (80.5)	44 (19.5)	2.26	(1.25 4.07)
1960–1969	238	56 (23.5)	182 (76.5)	2.38	(1.50 3.77)	234	156 (66.7)	78 (33.3)	5.45	(3.26 9.10)
1970–1979	101	31 (30.7)	70 (69.3)	1.68	(0.96 2.92)	99	64 (64.7)	35 (35.3)	4.98	(2.63 9.43)
1980–1989	31	15 (48.4)	16 (51.6)	0.77	(0.34 1.78)	30	23 (76.7)	7 (23.3)	2.05	(0.64 6.58)
Total	1144	326 (28.5)	818 (71.5)			1127	890 (79.0)	237 (21.0)		
*Per Cohort OR*				1.09	(0.98 1.21)				1.41	(1.25 1.59)
*P*_*trend*_				0.12					< 0.0001	
***Post-Diagnosis***^***b***^										
1910–1929	68	66 (97.1)	2 (2.9)	1.00	REF	70	68 (97.1)	2 (2.9)	1.00	REF
1930–1939	184	176 (95.7)	8 (4.3)			187	181 (96.8)	6 (3.2)		
1940–1949	281	265 (94.3)	16 (5.7)	2.08	(0.83 5.26)	286	270 (94.4)	16 (5.6)	1.57	(0.64 3.88)
1950–1959	226	199 (88.1)	27 (11.9)	4.40	(1.88 10.29)	233	222 (95.3)	11 (4.7)	1.33	(0.46 3.81)
1960–1969	224	189 (84.4)	35 (15.6)	6.13	(2.60 14.43)	240	228 (95.0)	12 (5.0)	1.34	(0.59 3.04)
1970–1979	95	81 (85.3)	14 (14.7)	5.80	(2.20 15.27)	102	97 (95.1)	5 (4.9)	1.24	(0.38 4.00)
1980–1989	27	20 (74.1)	7 (25.9)	10.53	(3.24 34.29)	31	29 (93.6)	2 (6.5)	1.61	(0.30 8.60)
Total	1105	996 (90.1)	109 (9.9)			1149	1095 (95.3)	54 (4.7)		
*Per Cohort OR*				1.53	(1.32 1.76)				1.03	(0.85 1.25)
*P*_*trend*_				< 0.0001					0.76	
*OR*_*post* vs *pre*_^*c*^				0.04	(0.04 0.05)				0.19	(0.14 0.25)
*P*_*heterogeneity of trend*_^*c*^				< 0.0001					0.008	

* Participants were only asked about ever use of sunbeds once and were not asked to recall sunbed use at anchor ages

^a^ Model adjusted for sex, phenotype factor variables, center geography, years unaffected and clustered by family

^b^ Model adjusted for sex, phenotype factor variables, center geography, and clustered by family

^c^ Model adjusted for repeated individual data clustered by family

### Sunbed use

Less than a third (*n* = 683, 28.6%) of respondents reported ever using sunbeds (Table 4). There was a trend of increasing odds of sunbed use with increasing birth cohort (per cohort OR = 1.44, 95%CI:1.28 to 1.62), although the odds ratio estimate for the 1980s cohort was less than the 1970s cohort. A similar trend was seen after stratifying by melanoma status. There was no difference in odds of sunbed use or heterogeneity in trend when comparing cases and non-cases. Cases were less likely to use sunbeds after diagnosis than before diagnosis (OR = 0.19, 95%CI: 0.14 to 0.25). The trend in odds ratios of sunbed use by birth cohort before diagnosis was similar to that observed overall (per cohort OR = 1.41, 95%CI:1.25 to 1.59), while there was no trend in sunbed use by birth cohort after diagnosis.

## Discussion

To our knowledge, this is the first study to investigate trends of sun-related behavior by birth cohort among individuals in melanoma-prone families. We found increased sunscreen use with increasing birth cohort, regardless of SPF; and although we saw a trend of increasing odds of sunburn until the 1950s birth cohort, it decreased thereafter. We found no trends of sun exposure across birth cohorts, but we did note a trend of increasing odds of sunbed use with increasing birth cohort.

In our analysis of birth cohort-specific trends in sun-related behaviors by life period, we found that most participants experienced a sunburn at 11 to 20 years old, a time frame in which we also saw a trend of increasing sun exposure with increasing birth cohort during holidays. Sunscreen use was also lower during childhood (10 years old or younger) and adolescence (11 to 20 years old) compared to adulthood (after 20 years old). Because childhood and adolescence are critical windows of exposure for melanoma risk [16, 17], future interventions should be targeted towards these age groups.

When comparing between melanoma cases and non-cases, we found no differences in sunscreen use, sunburns, sunbed use, and sun exposure overall, during weekdays and weekends. A previous study also found no differences in lifetime or childhood sun exposure between cases and non-cases from melanoma-prone families [32]. These similarities may be due to shared behavior within families. However, we observed that cases had greater average sun exposure during holidays than non-cases, which may support the importance of intermittent sun exposure in melanoma pathogenesis within melanoma-prone families. Future research may need to focus on measures of intermittent sun exposure, such as the frequency and duration of specific recreational and vacation activities [33], as drivers of individual melanoma risk among melanoma-prone families.

Previous studies reported that a personal melanoma diagnosis acts as the strongest catalyst in changing one’s sun-related behavior [10, 34, 35]. In our study, we found higher sunscreen use, lower sun exposure, lower odds of sunburn and sunbed use among cases after diagnosis than before. However, we found a trend of increasing risk of sunburn with increasing birth cohort after diagnosis – a possible target for further investigation and prevention strategies.

Although we observed increased sunbed use in more recent birth cohorts compared to older ones, sunbeds and indoor tanning have become increasingly regulated by governments, including outright bans and restricted use by minors [36, 37]. In the last two decades, sunbed use has trended downwards among adolescents and adults globally [36, 38, 39].

We did not ask participants about their beliefs and/or attitudes regarding sun-related behaviors in our sun-exposure questionnaire instrument. A preference for tanned skin has been shown to increase sun-seeking behavior and sunbed use, even among high-risk individuals [10]. We did collect information on whether participants used sunscreen to prolong their sun exposure (data not shown), and although 40% who used sunscreen reportedly never used it to prolong their sun exposure, one-third (33%) of individuals used sunscreen to prolong their sun exposure at least half of the time or more throughout their life. Although we did not collect information on perceived melanoma risk, a subset of GenoMEL participants (*n* = 312) previously completed an online survey in which 79% perceived a greater risk of developing melanoma (or another melanoma) compared to other people of the same sex, age and skin color [40]. Translating this perception into agency remains a challenge.

Limitations of our study include our inability to examine the number of sunburns and number of sunbed sessions as continuous outcomes in our analyses. Both variables were zero-inflated and highly skewed, and our attempts at more complex modeling resulted in non-convergence due to small sample sizes.

## Conclusions

Although our study and others have shown improvements in sun-protective behavior over time, melanoma primary prevention activities remain suboptimal with room for enhancement during childhood and adolescence. More strategies need to be devised to mitigate melanoma risk by encouraging increased sunscreen use, decreased sun exposure and sunbed use among individuals from melanoma-prone families, with consideration for targeted strategies for individuals younger than 20 years of age.

## Supplementary Information

**Additional file 1 **: **Module 1.** Residency calendar, demographics, phenotype, and history of melanoma.

**Additional file 2 **: **Module 2.** Personal sun exposure

## Data Availability

Data will be made available upon request. This study involved human subjects and to protect the privacy of study participants, data requests will be reviewed by the GenoMEL Steering Committee. Requests for data related to this publication should be directed to Dr. Peter Kanetsky at peter.kanetsky@moffitt.org.

## Abbreviations

AMD: Adjusted mean difference
CI: Confidence interval
OR: Odds ratio
SD: Standard deviation
*SPF*: Sun protection factor

## References

[1] Erdmann F, Lortet-Tieulent J, Schuz J, Zeeb H, Greinert R, Breitbart EW, Bray F (2013). International trends in the incidence of malignant melanoma 1953-2008--are recent generations at higher or lower risk?. Int J Cancer.

[2] Loria D, Abriata MG, Santoro F, Latorre C (2020). Cutaneous melanoma in Argentina: an analysis of its characteristics and regional differences. Ecancermedicalscience.

[3] Wainstein AJA, Duprat Neto JP, Enokihara MY, Brechtbuhl ER, Riccardi F, Landman G, de Melo AC, de Lima VV, Munhoz RR, Dunshee De Abranches Oliveira Santos Filho I (2020). Demographic, clinical, and pathologic features of patients with cutaneous melanoma: final analysis of the Brazilian melanoma group database. JCO Glob Oncol.

[4] Gandini S, Sera F, Cattaruzza MS, Pasquini P, Picconi O, Boyle P, Melchi CF (2005). Meta-analysis of risk factors for cutaneous melanoma: II. Sun exposure. Eur J Cancer.

[5] Heerfordt IM, Philipsen PA, Larsen BO, Wulf HC (2017). Long-term trend in sunscreen use among beachgoers in Denmark. Acta Derm Venereol.

[6] Koch S, Pettigrew S, Minto C, Slevin T, Strickland M, Lin C, Jalleh G (2017). Trends in sun-protection behaviour in Australian adults 2007-2012. Australas J Dermatol.

[7] Rodvall Y, Wahlgren CF, Wiklund K (2019). Future reduction of cutaneous malignant melanoma due to improved sun protection habits and decreased common melanocytic nevi density among Swedish children?: a follow-up from 2002 to 2012. Eur J Cancer.

[8] Tabbakh T, Volkov A, Wakefield M, Dobbinson S (2019). Implementation of the SunSmart program and population sun protection behaviour in Melbourne, Australia: results from cross-sectional summer surveys from 1987 to 2017. PLoS Med.

[9] Begg CB, Hummer A, Mujumdar U, Armstrong BK, Kricker A, Marrett LD, Millikan RC, Gruber SB, Anton-Culver H, Klotz JB (2004). Familial aggregation of melanoma risks in a large population-based sample of melanoma cases. Cancer Causes Control.

[10] Diao DY, Lee TK (2013). Sun-protective behaviors in populations at high risk for skin cancer. Psychol Res Behav Manag.

[11] Shuk E, Burkhalter JE, Baguer CF, Holland SM, Pinkhasik A, Brady MS, Coit DG, Ariyan CE, Hay JL (2012). Factors associated with inconsistent sun protection in first-degree relatives of melanoma survivors. Qual Health Res.

[12] Glenn BA, Lin T, Chang LC, Okada A, Wong WK, Glanz K, Bastani R (2015). Sun protection practices and sun exposure among children with a parental history of melanoma. Cancer Epidemiol Biomarkers Prev.

[13] Wu YP, Parsons BG, Mooney R, Aspinwall LG, Cloyes K, Hay JL, Kohlmann W, Grossman D, Leachman SA (2018). Barriers and facilitators to melanoma prevention and control behaviors among at-risk children. J Community Health.

[14] Koch S, Pettigrew S, Hollier LP, Slevin T, Strickland M, Minto C, Jalleh G, Lin C (2016). Trends in Australian adolescents' sun-protection behaviours: implications for health campaigns. Aust N Z J Public Health.

[15] Gorig T, Diehl K, Greinert R, Breitbart EW, Schneider S (2018). Prevalence of sun-protective behaviour and intentional sun tanning in German adolescents and adults: results of a nationwide telephone survey. J Eur Acad Dermatol Venereol.

[16] Whiteman DC, Whiteman CA, Green AC (2001). Childhood sun exposure as a risk factor for melanoma: a systematic review of epidemiologic studies. Cancer Causes Control.

[17] Green AC, Wallingford SC, McBride P (2011). Childhood exposure to ultraviolet radiation and harmful skin effects: epidemiological evidence. Prog Biophys Mol Biol.

[18] Fischer AH, Wang TS, Yenokyan G, Kang S, Chien AL (2016). Sunburn and sun-protective behaviors among adults with and without previous nonmelanoma skin cancer (NMSC): a population-based study. J Am Acad Dermatol.

[19] Savoye I, Olsen CM, Whiteman DC, Bijon A, Wald L, Dartois L, Clavel-Chapelon F, Boutron-Ruault MC, Kvaskoff M (2018). Patterns of ultraviolet radiation exposure and skin cancer risk: the E3N-SunExp study. J Epidemiol.

[20] Centers for Disease C, Prevention (2012). Sunburn and sun protective behaviors among adults aged 18-29 years--United States**,** 2000-2010. MMWR Morb Mortal Wkly Rep.

[21] Gavin A, Boyle R, Donnelly D, Donnelly C, Gordon S, McElwee G, O'Hagan A (2012). Trends in skin cancer knowledge, sun protection practices and behaviours in the Northern Ireland population. Eur J Public Health.

[22] Ghiasvand R, Lund E, Edvardsen K, Weiderpass E, Veierod MB (2015). Prevalence and trends of sunscreen use and sunburn among Norwegian women. Br J Dermatol.

[23] Jones SE, Saraiya M, Miyamoto J, Berkowitz Z (2012). Trends in sunscreen use among U.S. high school students**:** 1999-2009. J Adolesc Health.

[24] Makin JK, Warne CD, Dobbinson SJ, Wakefield MA, Hill DJ (2013). Population and age-group trends in weekend sun protection and sunburn over two decades of the SunSmart programme in Melbourne, Australia. Br J Dermatol.

[25] Volkov A, Dobbinson S, Wakefield M, Slevin T (2013). Seven-year trends in sun protection and sunburn among Australian adolescents and adults. Aust N Z J Public Health.

[26] Azzarello LM, Dessureault S, Jacobsen PB (2006). Sun-protective behavior among individuals with a family history of melanoma. Cancer Epidemiol Biomarkers Prev.

[27] Wu YP, Parsons BG, Aspinwall LG, Hay JL, Boucher KM, Caputo H, Mooney R, Grossman D, Leachman SA (2019). Parent and child perspectives on perceived barriers to child sun protection and their association with sun protection strategies among children of melanoma survivors. Pediatr Dermatol.

[28] Tripp MK, Peterson SK, Prokhorov AV, Shete SS, Lee JE, Gershenwald JE, Gritz ER (2016). Correlates of sun protection and sunburn in children of melanoma survivors. Am J Prev Med.

[29] Kricker A, Armstrong BK, Goumas C, Litchfield M, Begg CB, Hummer AJ, Marrett LD, Theis B, Millikan RC, Thomas N (2007). Ambient UV, personal sun exposure and risk of multiple primary melanomas. Cancer Causes Control.

[30] Kricker A, Vajdic CM, Armstrong BK (2005). Reliability and validity of a telephone questionnaire for estimating lifetime personal sun exposure in epidemiologic studies. Cancer Epidemiol Biomarkers Prev.

[31] Sunscreen: A History [https://www.nytimes.com/2010/06/24/fashion/24skinside.html]. Accessed 22 June 2020.

[32] Siskind V, Aitken J, Green A, Martin N (2002). Sun exposure and interaction with family history in risk of melanoma, Queensland, Australia. Int J Cancer.

[33] Elwood JM (1992). Melanoma and sun exposure: contrasts between intermittent and chronic exposure. World J Surg.

[34] Vogel RI, Strayer LG, Engelman L, Nelson HH, Blaes AH, Anderson KE, Lazovich D (2017). Sun exposure and protection behaviors among long-term melanoma survivors and population controls. Cancer Epidemiol Biomarkers Prev.

[35] Lai YC, Yew YW (2018). Sunburns and sun-protective behaviors after a diagnosis of melanoma. Skinmed.

[36] Rodriguez-Acevedo AJ, Green AC, Sinclair C, van Deventer E, Gordon LG (2020). Indoor tanning prevalence after the International Agency for Research on Cancer statement on carcinogenicity of artificial tanning devices: systematic review and meta-analysis. Br J Dermatol.

[37] Indoor Tanning Restrictions for Minors | A State-By-State Comparison [https://www.ncsl.org/research/health/indoor-tanning-restrictions.aspx]. Accessed 22 June 2020.

[38] Bowers JM, Geller AC, Schofield E, Li Y, Hay JL (2020). Indoor tanning trends among US adults, 2007-2018. Am J Public Health.

[39] Guy GP, Watson M, Seidenberg AB, Hartman AM, Holman DM, Perna F (2017). Trends in indoor tanning and its association with sunburn among US adults. J Am Acad Dermatol.

[40] Branstrom R, Kasparian NA, Affleck P, Tibben A, Chang YM, Azizi E, Baron-Epel O, Bergman W, Chan M, Davies J (2012). Perceptions of genetic research and testing among members of families with an increased risk of malignant melanoma. Eur J Cancer.

