# Supplementary material for: Birth cohort-specific trends of sun-related behaviors among individuals from an international consortium of melanoma-prone families
Source: BMC Public Health. 2021 Apr 23;21:692. doi: 10.1186/s12889-021-10424-5 (PMC8063451; doi:10.1186/s12889-021-10424-5)
Supplement: Supplementary file 1 — Additional file 1 : Module 1. Residency calendar, demographics, phenotype, and history of melanoma. [file 12889_2021_10424_MOESM1_ESM.pdf]

MELANOMA GENETICS CONSORTIUM FAMILY STUDY

MODULE 1

CONFIDENTIAL

PERSONAL RESIDENCE AND WORK CALENDAR

Thank you for being a willing participant in our study. To answer these questions we ask that you think back over your life about:

- places you've lived
- jobs you've had
- time you've spent outdoors
- brief details of your skin
- family history of illness

Please:

- fill this questionnaire out as soon as you receive it
- send the completed questionnaire back in the reply paid envelope to:

All information that you give will be kept strictly confidential and will be used only in the preparation of reports in which you will not be identified personally. Your personal details will be filed separately from your interview answers.

Thank you.

OFFICE USE ONLY

SITE: ☐☐

FAMILY ID: ☐☐☐☐☐

INDIVIDUAL ID: ☐☐☐☐☐☐

ADMINISTRATION: **Self Administered...........1**
Face to Face ....2 ***(Interviewer initials ________)***
Telephone........................3 ***(Interviewer initials ________)***

SECTION 1
PERSONAL RESIDENCE AND WORK CALENDAR INSTRUCTIONS

The information in this section is used to work out how much exposure to the sun you have had at different times of your life. See example entries below.

**(1) AGE:**	This column is filled in already.
**(2) YEAR:**	This is the year that you turned the age listed in **(1)**. Write the year and date you were born next to "Date of Birth", then continue down the page to fill in each year up to your age now.
**(3) WHERE LIVING:**	For each place that you have lived, write the town or suburb and the state, or the country (if overseas) - just once opposite the year you started to live there. If you lived in more than one place in any year, write down the place where you lived the longest in that year.
**(4) SCHOOL OR JOB:**	For each school or job, write down the name of your school or job - just once opposite the year you started there. If you went to more than one school or had more than one job in a year, write down the one you had the longest.
**(5) and (6) DAYS AT WORK:**	Next to each school or job, please write down how many days a week you usually were in school or worked during the warmer months and during the cooler months. If your job or school did not follow a weekly cycle, please explain, for example by writing "two weeks on and four days off".<br><br>During your teen years in the warmer months you may have been in school or in a vacation job. We want to know on average how many days a week you usually were in school or worked during the warmer months.<br><br>After leaving school you may have been in a job or at college. If you were at college, during the warmer months you may have been in college or in a vacation job. We want to know on average how many days a week you usually were in school or worked during the warmer months.

Note:	Southern Hemisphere	Northern Hemisphere
Warmer months	October to March	April to September
Cooler months	April to September	October to March

EXAMPLE

(1) AGE	(2) YEAR	(3) WHERE WERE YOU LIVING?	(4) NAME OF SCHOOL, COLLEGE, UNIVERSITY OR JOB TITLE AND PLACE OF WORK	(5) DAYS PER WEEK AT WORK OR SCHOOL DURING WARMER MONTHS (see below)	(6) DAYS PER WEEK AT WORK OR SCHOOL DURING COOLER MONTHS (see below)	TO BE COMPLETED BY OFFICE STAFF ONLY	
						Latitude	Longitude
17	1983	Miami, FL	Miami High School	5	5		
18	1984	Los Angeles, CA	UCLA	4	4		
19	1985			“	“		
20	1986			‘”	“		
21	1987			‘”	“		
22	1988	New York, NY	Laboratory Technician, Harlem Hospital	5	5		
23	1989	“	“	“	“		
24	1990	“	“	“	“		
25	1991	Albany, NY	Researcher, NY State Department of Health	5	5		
26	1992	“	“	“	“		
27	1993	“	“	“	“		

CALENDAR AGE BIRTH to 10 years

1. What is your gender?

Male 1

Female...2

(1) AGE	(2) YEAR	(3) WHERE WERE YOU LIVING?	(4) NAME OF SCHOOL, COLLEGE, UNIVERSITY OR JOB TITLE AND PLACE OF WORK	(5) DAYS PER WEEK AT WORK OR SCHOOL DURING WARMER MONTHS (see below)	(6) DAYS PER WEEK AT WORK OR SCHOOL DURING COOLER MONTHS (see below)	TO BE COMPLETED BY OFFICE STAFF ONLY Latitude	Longitude
**Date of Birth** ___/__/_____ DD/MM/YYYY							
1	19__ / 20__						
2	19__ / 20__						
3	19__ / 20__						
4	19__ / 20__						
5	19__ / 20__						
6	19__ / 20__						
7	19__ / 20__						
8	19__ / 20__						
9	19__ / 20__						
10	19__ / 20__						

CALENDAR AGE 11 to 25 YEARS

(1) AGE	(2) YEAR	(3) WHERE WERE YOU LIVING?	(4) NAME OF SCHOOL, COLLEGE, UNIVERSITY OR JOB TITLE AND PLACE OF WORK	(5) DAYS PER WEEK AT WORK OR SCHOOL DURING WARMER MONTHS (see below)	(6) DAYS PER WEEK AT WORK OR SCHOOL DURING COOLER MONTHS (see below)	TO BE COMPLETED BY OFFICE STAFF ONLY	
						Latitude	Longitude
11	19__ / 20__						
12	19__ / 20__						
13	19__ / 20__						
14	19__ / 20__						
15	19__ / 20__						
16	19__ / 20__						
17	19__ / 20__						
18	19__ / 20__						
19	19__ / 20__						
20	19__ / 20__						
21	19__ / 20__						
22	19__ / 20__						
23	19__ / 20__						
24	19__ / 20__						
25	19__ / 20__						

CALENDAR AGE 26 to 40 YEARS

(1) AGE	(2) YEAR	(3) WHERE WERE YOU LIVING?	(4) NAME OF SCHOOL, COLLEGE, UNIVERSITY OR JOB TITLE AND PLACE OF WORK	(5) DAYS PER WEEK AT WORK OR SCHOOL DURING WARMER MONTHS (see below)	(6) DAYS PER WEEK AT WORK OR SCHOOL DURING COOLER MONTHS (see below)	TO BE COMPLETED BY OFFICE STAFF ONLY	
						Latitude	Longitude
26	19__ / 20__						
27	19__ / 20__						
28	19__ / 20__						
29	19__ / 20__						
30	19__ / 20__						
31	19__ / 20__						
32	19__ / 20__						
33	19__ / 20__						
34	19__ / 20__						
35	19__ / 20__						
36	19__ / 20__						
37	19__ / 20__						
38	19__ / 20__						
39	19__ / 20__						
40	19__ / 20__						

CALENDAR AGE 41 to 55 YEARS

(1) AGE	(2) YEAR	(3) WHERE WERE YOU LIVING?	(4) NAME OF SCHOOL, COLLEGE, UNIVERSITY OR JOB TITLE AND PLACE OF WORK	(5) DAYS PER WEEK AT WORK OR SCHOOL DURING WARMER MONTHS (see below)	(6) DAYS PER WEEK AT WORK OR SCHOOL DURING COOLER MONTHS (see below)	TO BE COMPLETED BY OFFICE STAFF ONLY	
						Latitude	Longitude
41	19__ / 20__						
42	19__ / 20__						
43	19__ / 20__						
44	19__ / 20__						
45	19__ / 20__						
46	19__ / 20__						
47	19__ / 20__						
48	19__ / 20__						
49	19__ / 20__						
50	19__ / 20__						
51	19__ / 20__						
52	19__ / 20__						
53	19__ / 20__						
54	19__ / 20__						
55	19__ / 20__						

CALENDAR AGE 56 to 70 YEARS

(1) AGE	(2) YEAR	(3) WHERE WERE YOU LIVING?	(4) NAME OF SCHOOL, COLLEGE, UNIVERSITY OR JOB TITLE AND PLACE OF WORK	(5) DAYS PER WEEK AT WORK OR SCHOOL DURING WARMER MONTHS (see below)	(6) DAYS PER WEEK AT WORK OR SCHOOL DURING COOLER MONTHS (see below)	TO BE COMPLETED BY OFFICE STAFF ONLY	
						Latitude	Longitude
56	19__ / 20__						
57	19__ / 20__						
58	19__ / 20__						
59	19__ / 20__						
60	19__ / 20__						
61	19__ / 20__						
62	19__ / 20__						
63	19__ / 20__						
64	19__ / 20__						
65	19__ / 20__						
66	19__ / 20__						
67	19__ / 20__						
68	19__ / 20__						
69	19__ / 20__						
70	19__ / 20__						

CALENDAR AGE 70 to 80 YEARS

(1) AGE	(2) YEAR	(3) WHERE WERE YOU LIVING?	(4) NAME OF SCHOOL, COLLEGE, UNIVERSITY OR JOB TITLE AND PLACE OF WORK	(5) DAYS PER WEEK AT WORK OR SCHOOL DURING WARMER MONTHS (see below)	(6) DAYS PER WEEK AT WORK OR SCHOOL DURING COOLER MONTHS (see below)	TO BE COMPLETED BY OFFICE STAFF ONLY	
						Latitude	Longitude
71	19__ / 20__						
72	19__ / 20__						
73	19__ / 20__						
74	19__ / 20__						
75	19__ / 20__						
76	19__ / 20__						
77	19__ / 20__						
78	19__ / 20__						
79	19__ / 20__						
80	19__ / 20__						

Note:	Southern Hemisphere	Northern Hemisphere
Warmer months	October to March	April to September
Cooler months	April to September	October to March

SECTION 2
COLORING, SKIN TYPE AND MOLES

Please circle one answer for each question below.

2. Which color best describes your natural hair color at age 18?

Red (any shade of red including auburn or strawberry blonde) 1
Fair or Blonde 2
Brown 3
Black 4

3. Which color best describes the color of your eyes?

Blue 1
Brown or Black 3
Any other color (such as gray or hazel or green) 2

4. Which color type best describes your skin before tanning or on areas never exposed to the sun, such as the inside of your upper arm?

Very fair 1
Fair 2
Olive 3
Brown 4
Black 5
Other ***(please specify ___________________)*** 6

5. Which statement best describes what would happen if your skin were exposed to bright sunlight for the first time in summer for one hour in the middle of the day without any protection?

Get a severe sunburn with blistering? 1
Have a painful sunburn for a few days followed by peeling? 2
Get mildly sunburned followed by some tanning? 3
Go brown without any sunburn? 4

6. Which of the following best describes what would happen to your skin if it were repeatedly exposed to bright sunlight in summer without any protection?

Go very brown and deeply tanned 1
Get moderately tanned 2
Get mildly or occasionally tanned 3
Get no suntan at all or only get freckled 4

7. <u>In general</u>, how does your skin react to the sun?

Always burns, never tans 1
Usually burns, sometimes tans 2
Sometimes burns, usually tans 3
Never burns, always tans 4

Please look at the faces below. Each of the faces shows some degree of freckling, from none to many.

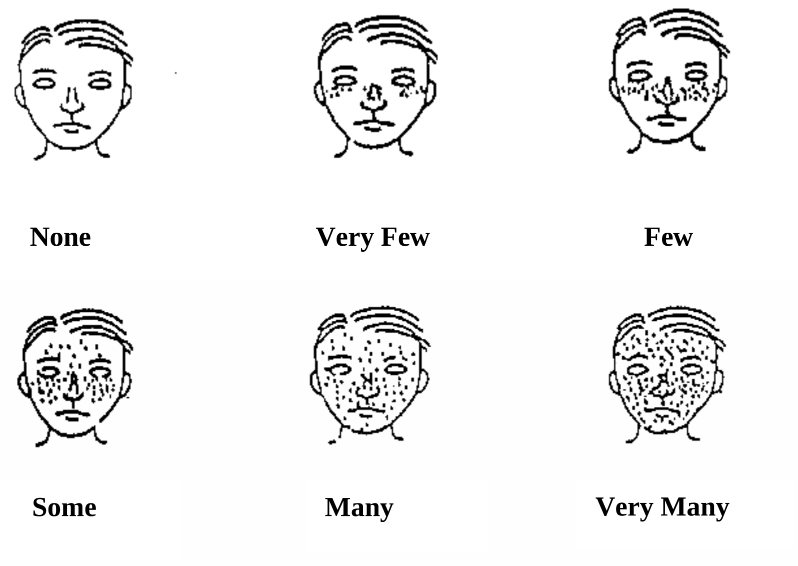

8. Which one of the above faces best describes how many freckles you would have had on your face at the end of summer <u>during childhood</u>?

None 1
Very few 2
Few 3
Some 4
Many 5
Very many 6

9. Which one of the above faces best describes how many freckles you would have on your face at the end of summer <u>as an adult</u>?

None 1
Very few 2
Few 3
Some 4
Many 5
Very many 6

Most people have some moles. Moles are brown spots that do not come and go with sunlight. Some are flat and look almost like a freckle while others are raised or bumpy if you run your fingers over them. Splotchy freckles on the upper back or shoulders are not moles. The color chart on the accompanying sheet showing different types of moles will help you decide what is a mole.

Please now look at the diagrams below that show various numbers of moles.

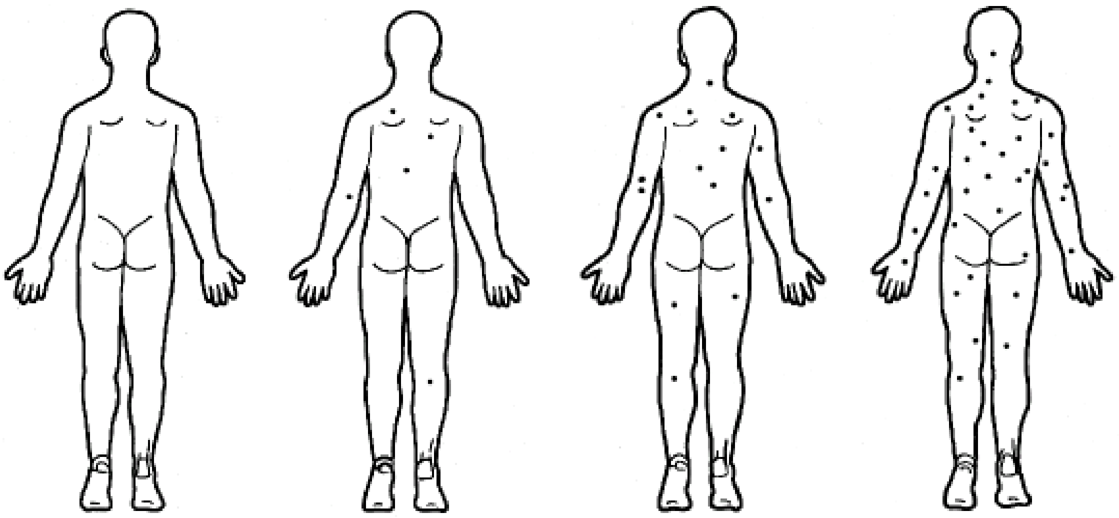

None **Few** **Some** **Many**

10. Which of these diagrams best describes how many moles you currently have?

None 1
Few 2
Some 3
Many 4

We would like to know the number of moles on your back. Please have someone count <u>all the moles on your back,</u> from the base of your neck to the top of your underpants. The area is shown within the rectangle of the diagram below. What is the <u>total number</u> of moles on your back from the base of your neck to the top of your underpants?

11.

Number of moles

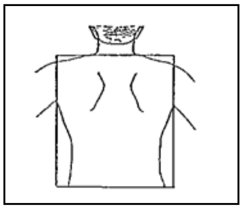

SECTION 3
FAMILY HISTORY OF CANCER

We would like to know if you or your family members have ever had cancer.

12. Have you ever had cancer?

Yes 1
No .... 2 **If your answer is 'No', please go to Question 17**
Don't Know 99

13. Have you ever been diagnosed with melanoma?

Yes 1
No .... 2 **If your answer is 'No', please go to Question 16**
Don't Know 99

14. Have you ever been diagnosed with more than one melanoma?

Yes 1
No 2
Don't Know 99

15. In what year(s) was/were the melanoma(s) diagnosed and at which hospital or physician's office was this diagnosed? If you require more space, please continue the table on the back of this page.

(Please include the hospital's or physician's complete name and address)

YEAR OF DIAGNOSIS	AGE AT THE TIME OF DIAGNOSIS	HOSPITAL OR PHYSICIANS NAME AND COMPLETE ADDRESS

16. **If you have had another type of cancer besides melanoma, where did it start? It is okay to report on other types of skin cancer.**

Please use the following numbered list to put the corresponding number in spaces provided below.
If you have had more than one type of cancer, additional spaces are provided. If you require more space, please continue on the back of this page.

1.	Adrenal	15.	Pancreas
2.	Bladder (urinary)	16.	Prostate
3.	Breast	17.	Sarcoma / Bone
4.	Cervix	18.	Skin, Basal Cell Carcinoma
5.	Colon / Rectum (bowel)	19.	Skin, Squamous Cell Carcinoma
6.	Esophagus	20.	Skin, Other (unsure of what type)
7.	Eye / Retinoblastoma	21.	Stomach
8.	Kidney (renal) / Wilms' tumor	22.	Testicle
9.	Leukemia	23.	Throat / Larynx
10.	Lung	24.	Thyroid / Parathyroid
11.	Lymphoma / Hodgkin's Disease	25.	Uterus (womb) / Endometrial
12.	Melanoma, ocular	26.	Brain
13.	Mouth	27.	Liver / Gall bladder
14.	Ovary		
		28.	If you are unsure how to classify a type of cancer or had a type that is not included in this table, please specify it here. (*please specify*) ________________________________

1st Cancer: Type ________________ Number _______ Year diagnosed: ______
2nd Cancer: Type ________________ Number _______ Year diagnosed: ______
3rd Cancer: Type ________________ Number _______ Year diagnosed: ______
4th Cancer: Type ________________ Number _______ Year diagnosed: ______

17. **Have any of the following people in your family had cancer?**
Please use the above numbered list from Question 16 to indicate specific cancer types.

				If yes:						
Relationship	Yes	No	Don't Know	First Cancer	Second Cancer	Age of diagnosis of first cancer	Has this person died? Yes	Has this person died? No	Current age if still alive	Age at death
(a) Spouse/Partner										
(b) Mother										
(c) Father										
(d) Mother's Mother										
(e) Mother's Father										
(f) Father's Mother										
(g) Father's Father										

Please answer questions 18 through 23 for <u>blood relatives only</u>, including half-brothers and half-sisters.

18. How many daughters and sons have you had? Please write 0 if you have not had any daughters/sons.

Number of daughters: _______

Number of sons: _______

19. How many brothers and sisters do you have or have you had? Please write 0 if you have not had any brothers/sisters.

Number of brothers: _______ Don't Know ☐ *(please check if appropriate)*

Number of sisters: _______ Don't Know ☐ *(please check if appropriate)*

20. How many aunts and uncles do you have or have you had on your mother's side? Please write 0 if you have not had any aunts/uncles.

Number of mother's sisters: _______ Don't Know ☐ *(please check if appropriate)*

Number of mother's brothers: _______ Don't Know ☐ *(please check if appropriate)*

21. How many aunts and uncles do you have or have you had on your father's side? Please write 0 if you have not had any aunts/uncles.

Number of father's sisters: _______ Don't Know ☐ *(please check if appropriate)*

Number of father's brothers: _______ Don't Know ☐ *(please check if appropriate)*

22. Have any of the family members mentioned above had cancer?
If yes, please use the numbered list from Question 16 to fill in the section below.
If no, please go to the next Question.

Relationship	First Cancer	Second Cancer	Age of diagnosis of first cancer	Has this person died?		Current age if still alive	Age at death
				Yes	No		
(a)							
(b)							
(c)							
(d)							
(e)							

23. Have any other blood relative members of your family not previously mentioned had cancer?

If yes, please use the numbered list from Question 16 to fill in the section below AND also indicate under the heading 'Relationship' which side of the family this relative is on (for example, mother's side).

If no, please go to the next Question.

Relationship	First Cancer	Second Cancer	Age of diagnosis of first cancer	Has this person died?		Current age if still alive	Age at death
				Yes	No		
(a)							
(b)							
(c)							
(d)							
(e)							

SECTION 4

ANCESTRY AND BODY MEASURES

24. Do you consider yourself to be Hispanic or Latino (of Spanish origin)?

Yes 1
No 2

25. Which of the following best describes you? ***Please select all that apply.***

White 1
Black or African American 2
Asian 3
Native Hawaiian or other Pacific Islander 4
American Indian or Alaska Native 5

Other (*please specify*) ____________________________

25b. Do you consider yourself to have a Jewish Heritage?

1. **No** _________
2. **Yes**, Ashkenazic (Eastern European Jewish) ____________
3. **Yes**, Sephardic (Southern European Jewish)_____________
4. **Yes**, Mixed, (Ashkenazic and Sephardic) _______________
5. **Yes,** Jewish (NOS) _________________
6. Do Not Know _________________

26. **In the table below, please check the choices that best describe the ethnic origins of your grandparents. Ethnic origin is based on a mixture of culture, religion, skin color, language, the origins of yourself and your family. It is not the same as nationality.**

Please select all that apply.

**Ethnic Origin**	**Mother's mother**	**Mother's father**	**Father's mother**	**Father's father**
**English** *(Originated from UK/Britain, England)*				
**Scottish, Irish, Manx or Welsh** *(Originated from Wales, Isle of Man, Scotland or Ireland)*				
**Other Northern European** *(Originated from Austria, Latvia, Lithuania, Estonia, Denmark, France, Germany, Luxembourg, Netherlands/Holland, Sweden, Norway, Finland, Switzerland, other Western/Northern European country)*				
**Southern European** *(Originated from Albania, Greece, Italy, Portugal, Spain, Former Yugoslavia, Malta, Cyprus, other Southern European country)*				
**Eastern European** *(Originated from Bulgaria, Former Czechoslovakia, Hungary, Poland, Romania, Former USSR, other Eastern European country)*				
**Indigenous Australian** *(Aboriginal, Torres Strait Islander)*				
**South-East Asian** *(Originated from Brunei, Cambodia, Indonesia, Laos, Malaysia, Myanmar/Burma, Philippines, Singapore, Thailand, Vietnam)*				
**North-East Asian** *(Originated from China, Hong Kong, Japan, Korea, Macau, Taiwan)*				
**South Asian** *(Originated from Afghanistan, Bangladesh, India, Nepal, Pakistan, Sri Lanka)*				
**Middle Eastern** *(Originated from Israel, Iran, Iraq, Lebanon, Turkey, Egypt or Arab)*				
**Pacific Islander** *(New Zealand Maori or originated from Pacific Islands, Hawaii, New Guinea)*				
**Indigenous American** *(American Indian)*				
**African-American** *(Person originating from North, Central, or South America, of Black African descent)*				
**American of Spanish or local "Indian" descent** *(Originating from North, Central or South America)*				
**Black African** *(Originating from North Africa, Sub-Saharan Africa, Zimbabwe or Black South African*				
**Other** (*please specify*) ________________________				

27. What is your height?

☐☐ . ☐☐

feet & inches

28. What is your weight now?

☐☐☐

pounds

29. Finally, what is the date that you completed this questionnaire?

☐☐ / ☐☐ / 20☐☐
M M D D Y Y

Thank you for your help with the Melanoma Genetic Consortium Family Study.

Please now place this form in the reply prepaid envelope and mail it back to us.
We will be in contact with you again soon.
