# Supplementary material for: Birth cohort-specific trends of sun-related behaviors among individuals from an international consortium of melanoma-prone families
Source: BMC Public Health. 2021 Apr 23;21:692. doi: 10.1186/s12889-021-10424-5 (PMC8063451; doi:10.1186/s12889-021-10424-5)
Supplement: Supplementary file 2 — Additional file 2 : Module 2. Personal sun exposure [file 12889_2021_10424_MOESM2_ESM.pdf]

MODULE 2

CONFIDENTIAL

PERSONAL INTERVIEW QUESTIONNAIRE

OFFICE USE ONLY

SITE: ☐☐

FAMILY ID: ☐☐☐☐☐

INDIVIDUAL ID: ☐☐☐☐☐☐

DATE OF BIRTH: ☐☐ / ☐☐ / ☐☐☐☐

DATE (Day/Month/Year): ☐☐ / ☐☐ /20☐☐

TIME INTERVIEW: Start ☐☐ ☐☐ am/pm

Finish ☐☐ ☐☐ am/pm

ADMINISTRATION: Face to Face .... 1 *(Interviewer initials ________)*
Telephone .... 2 *(Interviewer initials ________)*

PART A - SUN EXPOSURE

Enter appropriate data prior to interview where this marker appears.

If the subject reached one of the ages listed during the interview year, instructions should be about "last year". If the subject is aged between 50 and 70, the final questions are about “last year”.

SECTION A1 OUTDOOR ACTIVITIES

I want to thank you for agreeing to answer some questions about time you have spent outdoors.
I want to start by asking about the hours that you spent outdoors when you were 10 years old. By outdoors I mean outside and not under any shade.

(a) if the subject moved during a particular year, they should be asked to answer these questions for the place in which they spent the most time during that year.

(b) if the subject was away from home during a particular year, they should be asked to answer these questions for the next year when they were at home doing their usual activities.

At any time during Section 1, please remind the subject about the conditions for each Question if you feel they are uncertain, i.e. the age and in the warmer or cooler months

A - START QUESTIONS FOR 10 YEARS OF AGE

Let's begin with the year that you turned 10. That was in 19…... when you were living in ..………..……………………… *[town/suburb]* and going to ………………………………………..… school.

TIME OUTDOORS – WARMER MONTHS

Check Calendar Summary to determine where living at age 10
- *if living in southern hemisphere:* **Consider now only the warmer months, October to March.**
- *if living in northern hemisphere:* **Consider now only the warmer months, April to September.**

In that year,

A1 How many hours did you usually spend outdoors between 9 and 5 on school days?

Hours per day________ Don't Know ☐*

"Don't Know" is a last resort.
If the subject is uncertain, prompt with "Just give me the best estimate you can."

A2 How many hours did you usually spend outdoors between 9 and 5 on weekends?

Hours per day________ Don't Know ☐

A3 How many hours did you usually spend outdoors between 9 and 5 on school holidays?

Hours per day________ Don't Know ☐

TIME OUTDOORS –COOLER MONTHS

Check Calendar Summary to determine where living at age 10
- *if living in southern hemisphere:* **Now I want to ask about the cooler months, April to September.**
- *if living in northern hemisphere:* **Now I want to ask about the cooler months, October to March.**

In the year that you turned 10,

A4 How many hours did you usually spend outdoors between 9 and 5 on school days?

Hours per day________ Don't Know ☐

A5 And between 9 and 5 on weekends?

Hours per day________ Don't Know ☐

A6 And between 9 and 5 on school holidays?

Hours per day________ Don't Know ☐

SUMMER VACATIONS

A7 **Let's talk now about where you spent your summer vacations when you were 10, in 19…… when you were living in ....….…........…....... *[suburb/town]* and going to.……....…...............…................ school?**

A7.1 Vacation destination 1 (specify) ______________________

A7.2 Vacation destination 2 (specify) ______________________

Never went on holiday..2

Don't know..99

If subject answered 2 or 99, Skip to Question A12

I'm going to ask you a few questions about your holidays at *[Vacation destination 1 - from A7.1]*

A8 **How many weeks did you spend on summer vacations at this place?**

Weeks________ Don't Know ☐

A9 **How many hours a day between 9 and 5 did you usually spend outside in the sun during your summer vacation at this place?**

Hours ________ Don't Know ☐

If subject listed only one *VACATION DESTINATION* in Question A7, skip to Question A12

I'm now going to ask you a few questions about your holidays at *[Vacation destination 2 - from A7.2]*

A10 **How many weeks did you spend on summer vacations at this place?**

Weeks________ Don't Know ☐

A11 **How many hours a day between 9 and 5 did you usually spend outside in the sun during your summer vacation at this place?**

Hours________ Don't Know ☐

SUNSCREEN USE

A12 During this period when you were 10, how often did you use sunscreen lotions and creams to try to prevent sunburn when you were out in the sun?

Always or almost always 1
Not always but more than half the time 2
About half the time 3
Less than half the time 4
Never or hardly ever 5
Don't know 99

If subject answered 5 or 99, Skip to Question A15

A13 Was this usually a high protection sunscreen (high = SPF 8 or more)?

Yes 1
No 2
Don't know 99

A14 During this period, when you used sunscreen, how often did you use it to stay out in the sun longer?

Always or almost always 1
Not always but more than half the time 2
About half the time 3
Less than half the time 4
Never or hardly ever 5
Don't know 99

SUNBURN

A15 Were you ever sunburnt so as to cause pain for 2 or more days when you were 10 in 19...…. when you were living in ....…............... *[suburb/town]* and going to………................................... school?

Yes 1
No 2
Don't know 99

If subject answered 2 or 99, Skip to Question B1

A16 About how many times during the year did this happen?

Times________ Don’t Know ☐

A17 Were you ever sunburnt so severely during this year so as to cause blisters?

Yes 1
No 2
Don't know 99

If subject answered 2 or 99, Skip to B1

A18 About how many times during the year did this happen?

Times________ Don’t Know ☐

B - START QUESTIONS FOR 15 YEARS OF AGE

Let's talk now about the year that you turned 15. That was in 19..…. when you were living in…………………………. *[suburb/town]* and going to ……….……………………...………… school *or* working at ………………..……………………...

TIME OUTDOORS – WARMER MONTHS

Check Calendar Summary to determine where living at age 15

- *if living in southern hemisphere:* **Consider now only the warmer months, October to March.**
- *if living in northern hemisphere:* **Consider now only the warmer months, April to September.**

In that year,

B1 How many hours did you usually spend outdoors between 9 and 5 on school days?

Hours per day________ Don't Know ☐

B2 And between 9 and 5 on weekends or days off, not including holidays?

Hours per day________ Don't Know ☐

B3 And between 9 and 5 during school holidays?

Hours per day________ Don't Know ☐

TIME OUTDOORS – COOLER MONTHS

Check Calendar Summary to determine where living at age 15

- *if living in southern hemisphere:* **Now I want to ask about the cooler months, April to September.**
- *if living in northern hemisphere:* **Now I want to ask about the cooler months, October to March.**

In the year that you turned 15,

B4 How many hours did you usually spend outdoors between 9 and 5 on school days?

Hours per day________ Don't Know ☐

B5 And between 9 and 5 on weekends?

Hours per day________ Don't Know ☐

B6 And between 9 and 5 on school holidays?

Hours per day________ Don't Know ☐

SUMMER VACATIONS

B7 **Let's now talk about where you spent your summer vacations when you were 15, in 19...... when you were living in .... *[suburb/town]* and going to .... ……………school *or* working at ....………………..?**

B7.1 Vacation destination 1 (specify) ______________________

B7.2 Vacation destination 2 (specify) ______________________

Never went on holiday..2

Don't know..99

If subject answered 2 or 99, Skip to Question B12

I'm going to ask you a few questions about your holidays at *[* *Vacation destination 1 - from B7.1* ***]***

B8 **How many weeks did you spend on summer vacations at this place?**

Weeks________ Don't Know ☐

B9 **How many hours a day between 9 and 5 did you usually spend outside in the sun during your summer vacation at this place?**

Hours________ Don't Know ☐

If subject listed only one *VACATION DESTINATION* in Question B7, skip to Question B12

I'm now going to ask you a few questions about your holidays at *[Vacation destination 2 - from AB.2]*

B10 **How many weeks did you spend on summer vacations at this place?**

Weeks________ Don't Know ☐

B11 **How many hours a day between 9 and 5 did you usually spend outside in the sun during your summer vacation at this place?**

Hours________ Don't Know ☐

SUNSCREEN USE

B12 During this period when you were 15, how often did you use sunscreen lotions and creams to prevent sunburn when you were out in the sun?

Always or almost always 1
Not always but more than half the time 2
About half the time 3
Less than half the time 4
Never or hardly ever 5
Don't know 99

If subject answered 5 or 99, Skip to Question B15

B13 Was this usually a high protection sunscreen (high = SPF 8 or more)?

Yes 1
No 2
Don't know 99

B14 During this period, when you used sunscreen, how often did you use it to stay out in the sun longer?

Always or almost always 1
Not always but more than half the time 2
About half the time 3
Less than half the time 4
Never or hardly ever 5
Don't know 99

SUNBURN

B15 Were you ever sunburnt so as to cause pain for 2 or more days when you were 15 in 19...….. when you were living in .... *[suburb/town]* and going to ....……………........... school or working at ....………………?

Yes 1
No 2
Don't know 99

If subject answered 2 or 99, Skip to Question C1

B16 About how many times during the year did this happen?

Times________ Don't Know ☐

B17 Were you ever sunburnt so severely during this year so as to cause blisters?

Yes 1
No 2
Don't know 99

If subject answered 2 or 99, Skip to Question C1

B18 About how many times during the year did this happen?

Times________ Don't Know ☐

C - START QUESTIONS FOR 20 YEARS OF AGE

There will be some subjects who turn 20 during the year that they are interviewed. These individuals will be asked about the year that they turned 19.

Let's talk now about the year that you turned 19/20. That was in 19….... when you were living in ....……...........…….. *[suburb/town]* and you were going to ....…...………….................. college/university or working at ....……………….................…… job etc.

TIME OUTDOORS – WARMER MONTHS

Check Calendar Summary to determine where living at age 20

- *if living in southern hemisphere:* **Consider now only the warmer months, October to March.**
- *if living in northern hemisphere:* **Consider now only the warmer months, April to September.**

In that year,

C1 How many hours did you usually spend outdoors between 9 and 5 on weekdays?

Hours per day________ Don't Know ☐

C2 And between 9 and 5 on weekends or days off, not including holidays?

Hours per day________ Don't Know ☐

C3 *Only ask if subject was at college/university*

And between 9 and 5 during college/university holidays?

Hours per day________ Don't Know ☐

If subject responded to C3, Skip to Question C6

C4 *Only ask if subject was working*

Did you take time off for a week or more in the warmer months of the year that you turned 19/20?

Yes 1
No 2
Don't know.. 99

If subject answered 2 or 99, Skip to Question C6

C5 During this time off, how many hours did you usually spend outdoors between 9 and 5?

Hours per day_______ Don't Know ☐

TIME OUTDOORS – COOLER MONTHS

Check Calendar Summary to determine where living at age 20

- *if living in southern hemisphere:* **Now I want to ask about the cooler months, April to September.**
- *if living in northern hemisphere:* **Now I want to ask about the cooler months, October to March.**

In the year that you turned 20,

C6 How many hours did you usually spend outdoors between 9 and 5 on weekdays?

Hours per day________ Don't Know ☐

C7 **And between 9 and 5 on weekends?**

Hours per day________ Don't Know ☐

C8 *Only ask if subject is at college/university*

And between 9 and 5 during college/university holidays?

Hours per day________ Don't Know ☐

If subject responded to C8, Skip to Question C11

C9 *Only ask if subject is working*

Did you take time off for a week or more in the cooler months of the year that you turned 19/20?

Yes 1

No 2

Don't know 99

If subject answered 2 or 99, Skip to Question C11

C10 **During this time off, how many hours did you usually spend outdoors between 9 and 5?**

Hours per day________ Don't Know ☐

SUMMER VACATIONS

C11 **Let's talk now about where you spent your summer vacations when you were 19/20, in 19…. when you were living in ....*[suburb/town]* and going to .... college/university or working at ....………………………......... job etc?**

C11.1 Vacation destination 1 (specify) ___________________

C11.2 Vacation destination 2 (specify) ___________________

Never went on holiday 2

Don't know 99

If subject answered 2 or 99, Skip to Question C16

I'm going to ask you a few questions about your holidays at *[Vacation destination 1 - from C11.1]*

C12 **How many weeks did you spend on summer vacations at this place?**

Weeks ______________ Don't Know ☐

C13 **How many hours a day between 9 and 5 did you usually spend outside in the sun during your summer vacation at this place?**

Hours per day________ Don't Know ☐

If subject listed only one *VACATION DESTINATION* in Question C11, skip to Question C16

I'm now going to ask you a few questions about your holidays at *[Vacation destination 2 - from C11.2]*

C14 **How many weeks did you spend on summer vacations at this place?**

Weeks ______________ Don't Know ☐

C15 **How many hours a day between 9 and 5 did you usually spend outside in the sun during your summer vacation at this place?**

Hours per day________ Don't Know ☐

SUNSCREEN USE

C16 During this period when you were 19/20, how often did you use sunscreen lotions and creams to prevent sunburn when you were out in the sun?

Always or almost always 1
Not always but more than half the time 2
About half the time 3
Less than half the time 4
Never or hardly ever 5
Don't know 99

If subject answered 5 or 99, Skip to Question C19

C17 Was this usually a high protection sunscreen (high = SPF 8 or more)?

Yes 1
No 2
Don't know 99

C18 During this period, when you used sunscreen, how often did you use it to stay out in the sun longer?

Always or almost always 1
Not always but more than half the time 2
About half the time 3
Less than half the time 4
Never or hardly ever 5
Don't know 99

SUNBURN

C19 Were you ever sunburnt so as to cause pain for 2 or more days when you were 19/20 in 19….. when you were living in ....….. *[suburb/town]* and going to .....……...……………................ college/university or working at ....………........................ job etc?

Yes 1
No 2
Don't know 99

If subject answered 2 or 99, Skip to Question D1

C20 About how many times during the year did this happen?

Times ______________ Don't Know ☐

C21 Were you ever sunburnt so severely during this year so as to cause blisters?

Yes 1
No 2
Don't know 99

If subject answered 2 or 99, Skip to Question D1

C22 About how many times during the year did this happen?

Times ______________ Don't Know ☐

D - START QUESTIONS FOR 30 YEARS OF AGE

There will be some subjects who turn 30 during the year that they are interviewed. These individuals will be asked about the year that they turned 29.

Let's talk now about the year that you turned 29/30. That was in 19…... when you were living in ....…….........…….. *[suburb/town]* and you were going to ....…...…………................ college/university or working at ....………………................……. job etc.

TIME OUTDOORS – WARMER MONTHS

Check Calendar Summary to determine where living at age 30

- *if living in southern hemisphere:* **Consider now only the warmer months, October to March.**
- *if living in northern hemisphere:* **Consider now only the warmer months, April to September.**

In that year,

D1 How many hours did you usually spend outdoors between 9 and 5 on weekdays?

Hours per day________ Don't Know ☐

D2 And between 9 and 5 on weekends or days off, not including holidays?

Hours per day________ Don't Know ☐

D3 *Only ask if subject was at college/university*

And between 9 and 5 during college/university holidays?

Hours per day________ Don't Know ☐

If subject responded to D3, Skip to Question D6

D4 *Only ask if subject was working*

Did you take time off for a week or more in the warmer months of the year that you turned 29/30?

Yes 1
No 2
Don't know.. 99

If subject answered 2 or 99, Skip to Question D6

D5 During this time off, how many hours did you usually spend outdoors between 9 and 5?

Hours per day_______ Don't Know ☐

TIME OUTDOORS – COOLER MONTHS

Check Calendar Summary to determine where living at age 30

- *if living in southern hemisphere:* **Now I want to ask about the cooler months, April to September.**
- *if living in northern hemisphere:* **Now I want to ask about the cooler months, October to March.**

In the year that you turned 30,

D6 How many hours did you usually spend outdoors between 9 and 5 on weekdays?

Hours per day________ Don't Know ☐

D7 And between 9 and 5 on weekends?

Hours per day________ Don't Know ☐

D8 *Only ask if subject is at college/university*

And between 9 and 5 during college/university holidays?

Hours per day________ Don't Know ☐

If subject responded to D8, Skip to Question D11

D9 *Only ask if subject is working*

Did you take time off for a week or more in the cooler months of the year that you turned 29/30?

Yes 1

No 2

Don't know 99

If subject answered 2 or 99, Skip to Question D11

D10 During this time off, how many hours did you usually spend outdoors between 9 and 5?

Hours per day________ Don't Know ☐

SUMMER VACATIONS

D11 Let's talk now about where you spent your summer vacations when you were 29/30, in 19…. when you were living in ....*[suburb/town]* and going to .... college/university or working at ....……………………......... job etc?

D11.1 Vacation destination 1 (specify) ___________________

D11.2 Vacation destination 2 (specify) ___________________

Never went on holiday 2

Don't know 99

If subject answered 2 or 99, Skip to Question D16

I'm going to ask you a few questions about your holidays at *[Vacation destination 1 - from D11.1]*

D12 How many weeks did you spend on summer vacations at this place?

Weeks _____________ Don't Know ☐

D13 How many hours a day between 9 and 5 did you usually spend outside in the sun during your summer vacation at this place?

Hours per day ________________ Don't Know ☐

If subject listed only one *VACATION DESTINATION* in Question D11, skip to Question D16

I'm now going to ask you a few questions about your holidays at *[Vacation destination 2 - from D11.2]*

D14 How many weeks did you spend on summer vacations at this place?

Weeks _____________ Don't Know ☐

D15 How many hours a day between 9 and 5 did you usually spend outside in the sun during your summer vacation at this place?

Hours per day________ Don't Know ☐

SUNSCREEN USE

D16 During this period when you were 29/30, how often did you use sunscreen lotions and creams to prevent sunburn when you were out in the sun?

Always or almost always 1
Not always but more than half the time 2
About half the time 3
Less than half the time 4
Never or hardly ever 5
Don't know 99

If subject answered 5 or 99, Skip to Question D19

D17 Was this usually a high protection sunscreen (high = SPF 8 or more)?

Yes 1
No 2
Don't know 99

D18 During this period, when you used sunscreen, how often did you use it to stay out in the sun longer?

Always or almost always 1
Not always but more than half the time 2
About half the time 3
Less than half the time 4
Never or hardly ever 5
Don't know 99

SUNBURN

D19 Were you ever sunburnt so as to cause pain for 2 or more days when you were 29/30 in 19….. when you were living in ....….. *[suburb/town]* and going to .....……...……………................. college/university or working at ....……….......................... job etc?

Yes 1
No 2
Don't know 99

If subject answered 2 or 99, Skip to Question E1

D20 About how many times during the year did this happen?

Times ______________ Don't Know ☐

D21 Were you ever sunburnt so severely during this year so as to cause blisters?

Yes 1
No 2
Don't know 99

If subject answered 2 or 99, Skip to Question E1

D22 About how many times during the year did this happen?

Times ______________ Don't Know ☐

E - START QUESTIONS FOR 40 YEARS OF AGE

There will be some subjects who turn 40 during the year that they are interviewed. These individuals will be asked about the year that they turned 39.

Let's talk now about the year that you turned 39/40. That was in 19…... when you were living in ………………………………. *[suburb/town]* and you were going to ……………………………………… college/university or working at ………………………………………………. job etc.

TIME OUTDOORS – WARMER MONTHS

Check Calendar Summary to determine where living at age 40

- *if living in southern hemisphere:* **Consider now only the warmer months, October to March.**
- *if living in northern hemisphere:* **Consider now only the warmer months, April to September.**

In that year,

E1 How many hours did you usually spend outdoors between 9 and 5 on weekdays?

Hours per day________ Don't Know ☐

E2 And between 9 and 5 on weekends or days off, not including holidays?

Hours per day________ Don't Know ☐

E3 *Only ask if subject was at college/university*

And between 9 and 5 during college/university holidays?

Hours per day________ Don't Know ☐

If subject responded to E3, Skip to Question E6

E4 *Only ask if subject was working*

Did you take time off for a week or more in the warmer months of the year that you turned 39/40?

Yes 1
No 2
Don't know 99

If subject answered 2 or 99, Skip to Question E6

E5 During this time off, how many hours did you usually spend outdoors between 9 and 5?

Hours per day_______ Don't Know ☐

TIME OUTDOORS – COOLER MONTHS

Check Calendar Summary to determine where living at age 40

- *if living in southern hemisphere:* **Now I want to ask about the cooler months, April to September.**
- *if living in northern hemisphere:* **Now I want to ask about the cooler months, October to March.**

In the year that you turned 40,

E6 How many hours did you usually spend outdoors between 9 and 5 on weekdays?

Hours per day________ Don't Know ☐

E7 **And between 9 and 5 on weekends?**

Hours per day________ Don't Know ☐

E8 *Only ask if subject is at college/university*

And between 9 and 5 during college/university holidays?

Hours per day________ Don't Know ☐

If subject responded to E8, Skip to Question E11

E9 *Only ask if subject is working*

Did you take time off for a week or more in the cooler months of the year that you turned 39/40?

Yes... 1

No 2

Don't know... 99

If subject answered 2 or 99, Skip to Question E11

E10 **During this time off, how many hours did you usually spend outdoors between 9 and 5?**

Hours per day________ Don't Know ☐

SUMMER VACATIONS

E11 **Let's talk now about where you spent your summer vacations when you were 39/40, in 19…. when you were living in ....*[suburb/town]* and going to .... college/university or working at ....……………………......... job etc?**

E11.1 Vacation destination 1 (specify) ___________________

E11.2 Vacation destination 2 (specify) ___________________

Never went on holiday.. 2

Don't know... 99

If subject answered 2 or 99, Skip to Question E16

I'm going to ask you a few questions about your holidays at *[Vacation destination 1 - from E11.1]*

E12 **How many weeks did you spend on summer vacations at this place?**

Weeks _____________ Don't Know ☐

E13 **How many hours a day between 9 and 5 did you usually spend outside in the sun during your summer vacation at this place?**

Hours per day________ Don't Know ☐

If subject listed only one *VACATION DESTINATION* in Question E11, skip to Question E16

I'm now going to ask you a few questions about your holidays at *[Vacation destination 2 - from E11.2]*

E14 **How many weeks did you spend on summer vacations at this place?**

Weeks _____________ Don't Know ☐

E15 **How many hours a day between 9 and 5 did you usually spend outside in the sun during your summer vacation at this place?**

Hours per day________ Don't Know ☐

SUNSCREEN USE

E16 During this period when you were 39/40, how often did you use sunscreen lotions and creams to prevent sunburn when you were out in the sun?

Always or almost always 1
Not always but more than half the time 2
About half the time 3
Less than half the time 4
Never or hardly ever 5
Don't know.. 99

If subject answered 5 or 99, Skip to Question E19

E17 Was this usually a high protection sunscreen (high = SPF 8 or more)?

Yes 1
No 2
Don't know.. 99

E18 During this period, when you used sunscreen, how often did you use it to stay out in the sun longer?

Always or almost always 1
Not always but more than half the time 2
About half the time 3
Less than half the time 4
Never or hardly ever 5
Don't know.. 99

SUNBURN

E19 Were you ever sunburnt so as to cause pain for 2 or more days when you were 39/40 in 19….. when you were living in ....….. *[suburb/town]* and going to .....……...…………….................. college/university or working at ....……….......................... job etc?

Yes 1
No 2
Don't know.. 99

If subject answered 2 or 99, Skip to Question F1

E20 About how many times during the year did this happen?

Times ______________ Don’t Know ☐

E21 Were you ever sunburnt so severely during this year so as to cause blisters?

Yes 1
No 2
Don't know..99

If subject answered 2 or 99, Skip to Question F1

E22 About how many times during the year did this happen?

Times ______________ Don’t Know ☐

F - START QUESTIONS FOR LAST YEAR (SUBJECTS OVER 49 YEARS)

There will be some subjects who are over 49 years old. These individuals will be asked about the 'last' year.

Let's talk now about last year. That was in 20…..... when you were living in ....……...........…….. *[suburb/town]* and you were going to ....…....…………................ college/university or working at ....……………….................……. job etc.

TIME OUTDOORS – WARMER MONTHS

Check Calendar Summary to determine where living last year

- *if living in southern hemisphere:* **Consider now only the warmer months, October to March.**
- *if living in northern hemisphere:* **Consider now only the warmer months, April to September.**

Last year,

F1 How many hours did you usually spend outdoors between 9 and 5 on weekdays?

Hours per day________ Don't Know ☐

F2 And between 9 and 5 on weekends or days off, not including holidays?

Hours per day________ Don't Know ☐

F3 *Only ask if subject was at college/university*

And between 9 and 5 during college/university holidays?

Hours per day________ Don't Know ☐

If subject responded to F3, Skip to Question F6

F4 *Only ask if subject was working*

Did you take time off for a week or more in the warmer months of last year?

Yes 1
No 2
Don't know 99

If subject answered 2 or 99, Skip to Question F6

F5 During this time off, how many hours did you usually spend outdoors between 9 and 5?

Hours per day_______ Don't Know ☐

TIME OUTDOORS – COOLER MONTHS

Check Calendar Summary to determine where living last year

- *if living in southern hemisphere:* **Now I want to ask about the cooler months, April to September.**
- *if living in northern hemisphere:* **Now I want to ask about the cooler months, October to March.**

During last year,

F6 How many hours did you usually spend outdoors between 9 and 5 on weekdays?

Hours per day________ Don't Know ☐

F7 **And between 9 and 5 on weekends?**

Hours per day________ Don't Know ☐

F8 *Only ask if subject is at college/university*

And between 9 and 5 during college/university holidays?

Hours per day________ Don't Know ☐

If subject responded to F8, Skip to Question F11

F9 *Only ask if subject is working*

Did you take time off for a week or more in the cooler months of last year?

Yes 1
No 2
Don't know 99

If subject answered 2 or 99, Skip to Question F11

F10 **During this time off, how many hours did you usually spend outdoors between 9 and 5?**

Hours per day________ Don't Know ☐

SUMMER VACATIONS

F11 **Let's talk now about where you spent your summer vacations last year, in 20…. when you were living in .... *[suburb/town]* and going to .... college/university or working at .... job etc?**

F11.1 Vacation destination 1 (specify) ___________________

F11.2 Vacation destination 2 (specify) ___________________

Never went on holiday 2
Don't know 99

If subject answered 2 or 99, Skip to Question F16

I'm going to ask you a few questions about your holidays at *[Vacation destination 1 - from F11.1]*

F12 **How many weeks did you spend on summer vacations at this place?**

Weeks ______________ Don't Know ☐

F13 **How many hours a day between 9 and 5 did you usually spend outside in the sun during your summer vacation at this place?**

Hours per day________ Don't Know ☐

If subject listed only one *VACATION DESTINATION* in Question F11, skip to Question F16

I'm now going to ask you a few questions about your holidays at *[Vacation destination 2 - from F11.2]*

F14 **How many weeks did you spend on summer vacations at this place?**

Weeks ______________ Don't Know ☐

F15 **How many hours a day between 9 and 5 did you usually spend outside in the sun during your summer vacation at this place?**

Hours per day________ Don't Know ☐

SUNSCREEN USE

F16 During last year, how often did you use sunscreen lotions and creams to prevent sunburn when you were out in the sun?

Always or almost always 1
Not always but more than half the time 2
About half the time 3
Less than half the time 4
Never or hardly ever 5
Don't know 99

If subject answered 2 or 99, Skip to Question G1

If subject answered 5 or 99, Skip to Question F19

F17 Was this usually a high protection sunscreen (high = SPF 8 or more)?

Yes 1
No 2
Don't know 99

F18 During this period, when you used sunscreen, how often did you use it to stay out in the sun longer?

Always or almost always 1
Not always but more than half the time 2
About half the time 3
Less than half the time 4
Never or hardly ever 5
Don't know 99

SUNBURN

F19 Were you ever sunburnt so as to cause pain for 2 or more days last years in 20….. when you were living in ……………………….. *[suburb/town]* and going to …………………………………. college/university or working at ……………………………… job etc?

Yes 1
No 2
Don't know 99

If subject answered 2 or 99, Skip to Question G1

F20 About how many times during the year did this happen?

Times ______________ Don't Know ☐

F21 Were you ever sunburnt so severely during this year so as to cause blisters?

Yes 1
No 2
Don't know 99

If subject answered 2 or 99, Skip to Question G1

F22 About how many times during the year did this happen?

Times ______________ Don't Know ☐

G - SUNBEDS

Now I'd like to ask about whether you've ever used a sunlamp or tanning bed.

G1 Have you ever used a sunlamp or tanning bed for any reason on more than one occasion?

Yes 1
No 2
Don't know.. 99

If subject answered 2, interview is completed. Skip to Comment section.

G2 How old were you when you last used one?

Years old _____ Don't Know ☐

G3 How old were you when you first used one?

Years old _____ Don't Know ☐

G4 About how many sunlamp/tanning bed sessions have you had in total over your lifetime?

Sessions______ Don't Know ☐

That's all the questions for now.
Have you any comments, or information that you think we should have asked about?

....

....

....

....

....

....

....

....

....

....

Thank you for your patience and cooperation and for taking time out of your day to help us with this important research.
